# Supplementary material for: Projected impact of a reduction in sugar-sweetened beverage consumption on diabetes and cardiovascular disease in Argentina: A modeling study
Source: PLoS Med. 2020 Jul 28;17(7):e1003224. doi: 10.1371/journal.pmed.1003224 (PMC7386620; doi:10.1371/journal.pmed.1003224)
Supplement: S1 Appendix — Update and calibration of CVDPM-Argentina. SSB consumption estimations. Table A: Local data sources for CVD Policy Model-Argentina update and calibration. Table B: Comparison of overall outcomes between model predictions (CVDPM-Arg) and actual statistics in Argentina, 2010. Table C: Summary of variables used for the forecasting of the effect of SSB taxation in Argentina on diabetes, CVDs, and mortality outcomes. Table D: Daily per capita consumption of SSBs in Argentina, by age group and gender, by self-report from 2 cities, 2010–2011. Table E: Comparison of 2 different methodologies for estimating SSB per capita daily consumption in Argentina. CVD, cardiovascular disease; CVDPM-Arg, Cardiovascular Disease Policy Model-Argentina; SSB, sugar-sweetened beverage or soda. (DOCX) [file pmed.1003224.s001.docx]

**S1 Appendix: The Cardiovascular Disease Policy Model (CVDPM). Update and Calibration of CVDPM-Argentina. Sugar-sweetened beverages (SSB) consumption estimations.**

Contents

[Updated CVD Policy Model - Argentina 3](#_Toc43452672)

[Model Calibration 4](#_Toc43452673)

[Risk functions for incident diabetes, CHD, stroke and Non-CVD death 5](#_Toc43452674)

[Transition between risk factors 6](#_Toc43452675)

[Population Impact Fraction 7](#_Toc43452676)

[Simulation input assumptions 7](#_Toc43452677)

[Estimation of daily SSB consumption in Argentina 8](#_Toc43452678)

[Daily *per capita* sugar-sweetened soda consumption according to CESCAS I study 8](#_Toc43452679)

[Description of the alternative method used to estimate daily per capita sugar-sweetened soda consumption in Argentina, and comparison with CESCAS I study 8](#_Toc43452680)

[Appendix Figures 11](#_Toc43452681)

[S1 Figure: Cardiovascular Disease (CVD) Policy model structure 11](#_Toc43452682)

[S2 Figure. Directed acyclic graph describing the relationship between changes in SSB consumption and risk factors and outcomes in the CVD Policy Model –Argentina. 12](#_Toc43452683)

[Appendix Tables 13](#_Toc43452684)

[Table A: Local data sources for CVD policy model-Argentina update and calibration 13](#_Toc43452685)

[Table B: comparison of overall outcomes between model predictions (CVDPM-Arg) and actual statistics in Argentina, 2010 14](#_Toc43452686)

[Table C: Summary of variables used for the forecasting of the effect of SSB taxation in Argentina on diabetes, cardiovascular diseases, and mortality outcomes 15](#_Toc43452687)

[Table D: Daily *per capita* consumption of sugar sweetened sodas in Argentina, by age group and gender by self-report from 2 cities, 2010-2011 16](#_Toc43452688)

[Table E: comparison of two different methodologies for estimating sugar-sweetened sodas *per capita* daily consumption in Argentina 17](#_Toc43452689)

[References 18](#_Toc43452690)

**Model Overview**

The CVD Policy model is a computer simulation, state transition (Markov cohort) model that estimates the prevalence and incidence of cardiovascular disease, as well as its related mortality and direct associated costs, by using epidemiological data of the population 35 years old and older (S1 Figure). The CVDPM-Argentina is a version of the CVD Policy Model that uses Argentina-specific inputs wherever possible and is calibrated within <1% of the total number of CVD events and deaths in 2010 as reported in national data. The model consists of 3 sub-models: the demographic-epidemiologic submodel (DE), which includes the population without CVD and predicts type 2 diabetes, coronary heart disease (CHD) and stroke incidence, and non-CVD mortality; the bridge submodel, which characterizes the initial CVD event and its sequelae for 30 days; and the disease-history submodel (DH), which estimates subsequent CVD events in people with previous CVD (S1 Figure).[1,2]

The CVD Policy Model-Argentina incorporates 6 risk factors: systolic blood pressure (SBP), low-density lipoprotein cholesterol (LDL-c), high-density lipoprotein cholesterol (HDL-c), smoking, diabetes, and body mass index (BMI); each of which are represented by their mean and prevalence for 3 predefined categories, stratified by gender (male or female) and 10-year age group (from 35-44 years old through 85-94 years old):

- SBP: <130; 130-139.9; ≥ 140 mmHg
- LDL-c: < 100; 100-129.9; ≥130 mg/dl
- HDL-c: <40; 40-59.9; ≥ 60 mg/dl
- Smoking: active, passive, and non-smoking
- Diabetes: yes or no
- BMI: <25; 25-29.9; ≥ 30 kg/m^2^

The model can be used to forecast CVD trends and simulate interventions through the modification of input data including population structure and dynamics, risk factor distributions, coefficients defining the relationship between risk factors and disease incidence, event and case fatality rates, costs, and quality of life adjustments. All adults 35 to 94 years of age who are alive in a given simulation year are distributed into healthy (non-CVD) or CVD states; the non-CVD population is further stratified according to diabetes status. The risk reduction associated with simulated improvements in one or more risk factors is calculated for each cell based on age, gender, and baseline mean risk factor values. Each simulation runs on an annual basis and outcomes change over time according to the dynamics of the population’s demographic shifts and risk factor distributions.[1,3]

Each year, based on the multivariate distribution of risk factors, the model estimates the fraction of the diabetes-free population that develops type 2 diabetes as well as the fraction of the CVD-free population that develops coronary heart disease or stroke. The subpopulation experiencing an incident coronary heart disease (angina, myocardial infarction (MI), or cardiac arrest) or stroke event moves into the bridge submodel, where the initial event is characterized along with its sequelae over the first 30 days (including recurrent MI, stroke, arrest, and revascularization procedures). The disease-history submodel estimates subsequent MI and stroke events, and CVD and non-CVD mortality in the population with a history of CVD, stratified by prior CVD history, age group and sex. The CVD states include angina only, MI only, stroke only, and Stroke + MI. Each year, there is a probability of having a repeat event and/or transitioning to a new CVD state.

# Updated CVD Policy Model - Argentina

The original version of Argentina’s CVD Policy Model was developed in 2009. Since then, new sources of information have become available including the following:

- The National Census conducted in 2010 was used to update the model to reflect the 2010 Argentine population and to estimate 35 years-olds entering the modeled population each year from 2011 to 2100.[4-6]
- The 2013 National Risk Factor Survey, a national representative survey of Argentine population 18 years and older living in private homes in urban areas of 5,000 and more inhabitants, was used to update age and sex-specific means and distributions for BMI, smoking and diabetes.[7]
- The “Study for the detection and follow up of cardiovascular disease risk factors in the southern cone of Latin America”, coordinated through the Centro de Excelencia en Salud Cardiovascular para América del Sur (CESCAS I study), an on-going observational prospective cohort designed to study cardiovascular disease prevalence and risk factors in Southern Latin America, provided information on age and sex specific means and prevalence for LDL-c, HDL-c and SBP.[8] These variables were not measured in the 2013 National Risk Factor Survey.
- The Program for the epidemiological evaluation of stroke in Tandil (PrEViSTA) study, which reports local information on First-Ever Stroke and Transient Ischemic Attack Incidence, was used to update age- and sex-stratified stroke incidence rates.[9]

## Model Calibration

After risk factors and demographic inputs were updated, the model was calibrated to reproduce contemporary cardiovascular disease morbidity and mortality data.

Argentina’s population and risk factor distributions determined the rate of incident cardiovascular events (first events in the population without disease). We gathered local information on the number of CVD events (CHD and strokes) as well as CVD mortality for the year 2010 to use as standards for calibrating model transition rates. CVD, non-CVD, and total deaths (by age and gender) per year were estimated from Argentina’s National Vital Statistics for the year 2010. The actual total number of deaths attributable to coronary heart disease was estimated as a compound of both definite CHD deaths (codes I20-I25 of the International Classification of Diseases, 10th Revision [10]) in health records plus a percentage of poorly defined deaths (named ‘garbage’ codes, already defined) that could be attributed to CHD deaths.[11,12] Garbage codes are codes assigned to deaths that were supposedly misclassified, a percentage of which belong actually to CHD deaths.[11,12] The total number of deaths obtained was later corrected by a factor determined for countries with low quality of registry (such as Argentina) in the Global Burden of Disease initiative.[13] A similar method was then used to compare predicted and reported stroke deaths (codes I60-I69).

Due to the absence of national data on the total number of myocardial infarction, arrest, or stroke events occurring annually in each age and gender group, we used data from local studies including the Sindromes Coronarios Agudos en Argentina study [14], a multicenter registry of CHD events in Argentina, and the PrEViSTA Study, in conjunction with US event rates, in order to infer annual targets used to calibrate the Argentina model.

Initial event rates in the population with prevalent cardiovascular disease were assumed from the prior model version. For calibration of mortality outcomes, the actual total number of deaths attributable to coronary heart disease was estimated as a compound of both definite CHD deaths (codes I20-I25 of the International Classification of Diseases, 10th Revision [10]) in health records plus a percentage of poorly defined deaths (named ‘garbage’ codes, already defined) that could be attributed to CHD deaths.[11,12] Garbage codes are codes assigned to deaths that were supposedly misclassified as non-CHD deaths when they should have been coded as CHD deaths. The total number of deaths obtained was later corrected by a factor determined for countries with low quality of registry (such as Argentina) in the Global Burden of Disease initiative.[13] A similar method was then used to compare predicted and reported stroke deaths (using codes I60-I69). Model predictions were then adjusted iteratively until they matched the actual health statistics in 2010, the baseline year (Table B in S1 Appendix).

After the process of calibration, real and simulated total number of events and deaths due to MIs, arrest or strokes were compared, as well as total and non-CVD mortality.

In all cases, the final version of the model predicted the actual number of events with an accuracy superior to 99.5%.

A list of the local data sources used for the update and calibration of the CVD policy model-Argentina can be seen in Table A in S1 Appendix.

A more detailed description of the update and calibration process can be found in a previous publication.[15]

# Risk functions for incident diabetes, CHD, stroke and Non-CVD death

The incidence of outcomes (i.e., type 2 diabetes, CHD, stroke, and non-CVD death) in each risk factor cell for the at-risk population were determined by a risk function incorporating age- and sex-specific alphas, risk factor-specific betas (which are constant over the time span of a simulation), and cell-specific risk factor means (which are altered by user-defined interventions). The risk function is defined as:

$$r=e^{(\alpha+\sum_{k=1}^{6} \beta_{k}m_{k})}/(1+e^{(\alpha+\sum_{k=1}^{6} \beta_{k}m_{k})})$$

β-coefficients for incident CHD, incident stroke and non-CVD death were estimated using data from the Framingham Heart Study Original Cohort (exam 13-28) and Offspring Cohort (exam 1-7) [16-19] and the counting process extension of the Cox proportional hazards model, which allows for time-dependent covariates.[20] The diabetes incidence function is also estimated using Framingham data, with adjustments for the association between BMI and age based on published data demonstrating a lower effect of BMI on diabetes in older ages.[21] Multivariate models for incident CHD included as covariates SBP, LDL-c, smoking, HDL-c, and diabetes; models for stroke and for non-CVD death included SBP, smoking, and diabetes. Models for incident type 2 diabetes include only BMI. Coefficients generated from Framingham data have been reported to be useful in different populations.[22-24] The risk functions are applied to every state in every year of a simulation to accommodate the competing risk for all outcomes naturally over time. We assumed that all the risk factors modify the incidence of angina, infarction and arrest equally, except for smoking. Regarding excess risk from tobacco, we assumed that smokers had a higher risk for arrest and myocardial infarction and a lower excess risk for angina.[25] Environmental tobacco exposure had a 1.26 relative risk compared to non-exposed non-smokers just for cardiac arrest and myocardial infarction.[26]

# Transition between risk factors

We included annual probabilities of transition from one risk factor stratum to another to maintain age- and gender-specific risk factor proportions of the population as describe in CESCAS I study and the National Risk Factor Survey 2013.[7,8] The annual transition probabilities were calculated using an iterative algorithm that takes into account the competing risk effect from the model’s CVD incidence and non-CVD death rates established in the base case.

# Population Impact Fraction

We estimated the number of new cases of diabetes that would be prevented if current levels of SSB consumption are lowered by a percentage expected from the implementation of a national soda tax.[27] Percentage reductions in SSB intake were translated into absolute changes in risk factor means for each age and gender stratum, with status quo consumption calculated using data from CESCAS I study and Euromonitor (Appendix 2).[28,29] The resulting value comparing incident diabetes in the hypothetical intervention to that of the base case is known as the population impact fraction (PIF), defined as [30,31]:

$$PIF=\frac{\int_{i=1}^{max} PiRRi-\int_{i=1}^{max} Pi^{'}RRi}{\int_{i=1}^{Max} P\text{i}{RR}_{i}}$$

where i corresponds to various SSB consumption values as continuous variable; RRi is the fitted relative risk for developing diabetes at exposure level i, Pi is the base SSB intake distribution; and Pi’ represents the post reduction in consumption distribution. The resulting PIF therefore represents the relative reduction in diabetes incidence as a result of the percentage reduction in SSB intake intervention scenario.

# Simulation input assumptions

Expanding on information in the main manuscript, Table C in S1 Appendix presents a detailed list of variables and assumptions used for simulations forecasting the impact of SSB taxation in Argentina. The assumed relationship between changes in SSB consumption and changes in cardiovascular disease risk factors and outcomes are further depicted in Appendix S2 Figure.

## Estimation of daily SSB consumption in Argentina

We generated two estimates of SSB consumption for Argentine adults. Our “low estimate” was calculated directly from CESCAS I study data. We then attempted to validate CESCAS SSB daily consumption information by comparing it to estimates available from Euromonitor, an international data base on beverage sales.[29]

### Daily *per capita* sugar-sweetened soda consumption according to CESCAS I study

We obtained our “low” estimates from the CESCAS I study (Table D in S1 Appendix). In 2010-2011, CESCAS I measured self-reported average daily SSB consumption among 3300 adults aged 35 to 74 years old in two Argentinean cities (Bariloche and Marcos Paz).[8] We assumed consumption among people 75 years of age and older was equivalent to that reported by those 65 to 74 years old.

### Description of the alternative method used to estimate daily per capita sugar-sweetened soda consumption in Argentina, and comparison with CESCAS I study

For our “high” estimate of SSB consumption, we took CESCAS data, multiplied it by the population in each age group in 2010, annualized it, and thus estimated that the consumption of SSB among people older than 34 years old that year was 850.2 million liters. This would amount to 17.7% of 4810.9 million liters in SSB sold that year in Argentina per Euromonitor reports.[29] This rough calculation suggests that the CESCAS consumption numbers are either significantly under-reported or not representative of the country as a whole, as adults generally consume a larger proportion of SSB. For example, in Chile, a neighboring country with similar beverage consumption habits, the population aged 35 years and older consumes 41.5% of all SSBs.[32,33]

An alternate method to estimate sugary sodas consumption was then developed, using information from:

- Euromonitor (2015): data obtained from 2015 overall sales reports in Argentina.[29]
- National Nutrition and Health Survey (ENNyS): Cross sectional national coverage survey conducted in 2005 among women aged 10-49 years old. Does not differentiate between regular and diet soda.[34]
- CESCAS I study: It was used to stablished a ratio of consumption between different ages and gender.[8]

1. Using Euromonitor data, we estimated the percentage of the overall consumption of sodas that was attributable to regular (non-diet) sodas in 2005 (the year in which ENNyS was conducted) to be 87.1%.
2. We then compared, again using Euromonitor data, the overall consumption of regular SSBs in 2005 and 2015. Adjusting for the difference in population in both years, 2005 consumption represents 134.97% of 2015 consumption.
3. We developed the following approach to estimate daily serving size consumed in Argentina in 2015:
   1. We use the mean daily consumption in ENNyS as reference for women 35-44 .years old in 2005 (417.7 ml/day, including regular and diet SSB)

- 417.7 ml/day 🡪 363.82 ml/day of regular (no-diet) soda in 2005 (87.1% of 417.7 ml)
  1. We estimate the daily *per capita* volume consumed of regular soda in 2015:
- 363.82 ml/day of regular soda in 2005 (134.97%) 🡪 269.6 ml/day of regular soda in 2015 (100%) (corrected by the difference in population size in both years)
- 269.6 ml/day (9.12 oz/day) represents 0.76 of a 12 oz serving size per day in 2015 (women 35-44 .years old)
  1. We used CESCAS I proportion of consumption among genders and age groups to calculate the consumption for the rest of the age groups

Results can be seen in Table E in S1 Appendix. The difference in daily consumption obtained by the two methods was 210%.

# Appendix Figures





S1 Figure: Cardiovascular Disease (CVD) Policy model structure.

The CVD Policy Model is a state-transition simulation model of CVD in adults. State transitions are numbered in the diagram: Transition 1 = remain in CVD-free state, with 1-year cycle advancement of age and risk factor distribution; Transition 2 = incident CVD; Transition 3 = non-CVD death; Transitions 4 and 5 = survival or case fatality; Transition 6 = survival with or without repeat CVD event in chronic CVD patients. LDL = low density lipoprotein cholesterol; HDL = high density lipoprotein cholesterol; BMI = body mass index; MI = myocardial infarction.


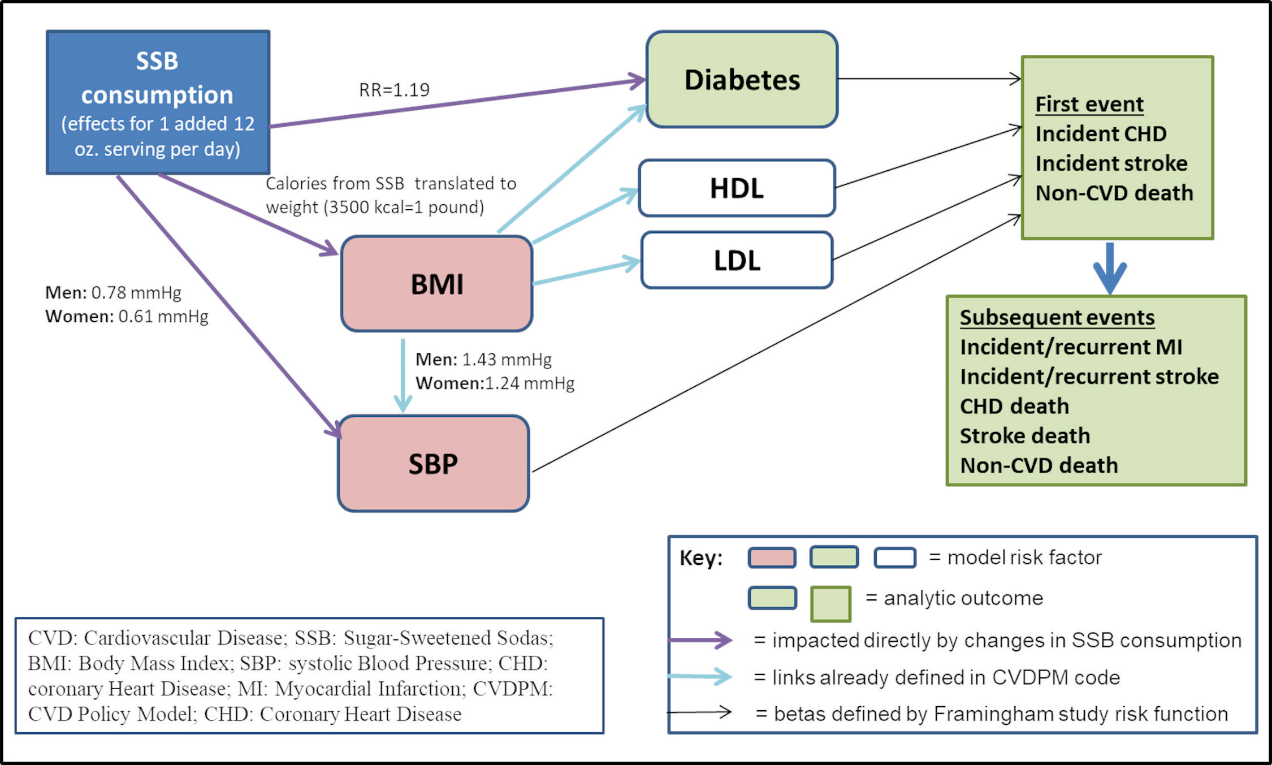


## S2 Figure. Directed acyclic graph describing the relationship between changes in SSB consumption and risk factors and outcomes in the CVD Policy Model –Argentina.

For model simulations, changes in SSB consumption were assumed to have direct and independent effects on model risk factors including BMI, SBP, and type 2 diabetes as well as indirect effects on SBP, diabetes, HDL and LDL that were mediated through BMI. Inputs for translating changes in SBP into changes in risk factors were based on published literature (see Table C in S1 Appendix). Framingham Heart Study data were used to determine the risk functions defining the relationships between BMI and diabetes as well as all risk factors and CVD and mortality outcomes. In this analysis (and in the CVD Policy Model in general), type 2 diabetes is both a risk factor connected to downstream CVD events and deaths as well as an outcome in and of itself. A detailed depiction of the CVD Policy Model – Argentina is shown in S1 Figure.

# Appendix Tables

| **Table A**: Local data sources for CVD policy model-Argentina update and calibration | |
| --- | --- |
| **Information** | **Source** |
| 2010 Argentina’s Population and Projections | 2010 National Census, Argentina National Statistics and Census Institute [4-6] |
| CVD Risk Factors (mean values and prevalences) | National Risk Factors Survey 2013 [7] and CESCAS I study [8,35] |
| MI Prevalence | National Risk Factors Survey 2013 [7] |
| Stroke Incidence | PREVISTA Study [9] |
| CVD and Non-CVD mortality | Argentina National Statistics and Census Institute [36] |
| Total number of MIs in 2010 | SCAR study (Sindromes Coronarios Agudos en Argentina) [14] |
| Total number of Strokes in 2010 | PREVISTA Study [9] |
| CVD: cardiovascular Disease; MI: myocardial infarction. | |

| **Table B**: comparison of overall outcomes between model predictions (CVDPM-Arg) and actual statistics in Argentina, 2010 | | | |
| --- | --- | --- | --- |
|  | **Real statistics**  **(n)** | **Model Predictions**  **(n)** | **Difference**  **(%)** |
| Total number of Mis | 41219 | 41265 | **0.11** |
| Total number of arrests | 10122 | 10132 | **0.10** |
| Total number of Strokes | 58658 | 58584 | **-0.13** |
| MI Deaths | 5354 | 5359 | **0.09** |
| Arrest Deaths | 10122 | 10132 | **0.10** |
| Stroke Deaths | 18241 | 18253 | **0.07** |
| Non-CVD Deaths | 231396 | 230391 | **-0.43** |
| Total deaths | 281710 | 280707 | **-0.36** |
| CVDPM-Arg: Cardiovascular Disease Policy Model – Argentina; MI: myocardial infarction; CVD: cardiovascular disease | | | |

| Table C: Summary of variables used for the forecasting of the effect of SSB taxation in Argentina on diabetes, cardiovascular diseases, and mortality outcomes | | |
| --- | --- | --- |
| **Variable** | **Input estimate** | **Data source** |
| Consumer Price Elasticity | -1.12 | Fernandez et al[27] |
| Baseline average intake of SSB by age group and gender  (ml/day) |  | Estimated from self-reported daily SSB consumption from CESCAS I study and Euromonitor sales report. [28,29] |
| Beverage caloric compensation by a SSB reduction in consumption | 39% | Assuming this percentage is composed by water (1/3), milk or juices (1/3), and diet beverages (1/3) [37] |
| Relative risk (RR) of excess SSB consumption on incident diabetes | 1.19 | Imamura et al. (2015) [38]; published values adjusted for definition of serving size |
| Beta for the association between a 1-unit increase in BMI and incident diabetes | Decreases over age;  Beta=0.161 for 55-64 year olds | Framingham Heart Study data [16-19]  Biggs et al. (2010) [21] |
| Changes in systolic blood pressure due to a reduction in SSB consumption of 1 serving/day | *Men: -*0.78 mmHg  *Women:* -0.61 mmHg | Chen L et al. [39] |
| Changes in systolic blood pressure per 1 unit increase in BMI* | *Men*: 1.43 mmHg  *Women:* 1.24 mm Hg | Wilsgaard et al.[40] |
| Changes in LDL per 1 unit increase in BMI | *Men:* 2.75 mg/dL  *Women*: 2.24 mg/dL | Wilsgaard et al.[41] |
| Changes in HDL per 1 unit increase in BMI | *Men:* 1.55 mg/dL  *Women*: 0.77 mg/dL | Wilsgaard et al.[41] |
| Increased risk of CHD associated with diabetes** | RR=1.84 | Framingham Heart Study data [16-19] |
| Beta for the association of SBP and incident CHD** | Decreases over age; beta=0.013 for 60 year old | Framingham Heart Study data [16-19] |
| Increased risk of stroke associated with diabetes*** | RR=2.31 | Framingham Heart Study data [16-19] |
| Beta for the association of SBP with incident stroke*** | Decreases over age; beta=0.019 for 60 year old | Framingham Heart Study data [16-19] |
| Increased risk of non-CVD mortality associated with diabetes**** | Decreases over age;  RR=1.57 for 60 year old | Framingham Heart Study data [16-19] |
| Beta for the association of SBP with non-CVD mortality**** | Decreases over age;  Beta=0.00468 for 60 year old | Framingham Heart Study data [16-19] |
| **Abbreviations:** CHD: coronary heart disease; CVD: cardiovascular disease; SBP: systolic blood pressure; LDL: Low-density lipoprotein cholesterol; HDL: high-density lipoprotein cholesterol  ** Models included the following variables: diabetes, SBP, LDL,HDL, and smoking; assumed age interaction for SBP and diabetes  *** Models included the following variables: SBP, smoking and diabetes; assumed age interactions with SBP  **** Models included the following variables: SBP, smoking and diabetes; assumed age interactions with SBP and diabetes | | |

| **Table D**: Daily *per capita* consumption of sugar sweetened sodas in Argentina, by age group and gender by self-report from 2 cities, 2010-2011 | | | |
| --- | --- | --- | --- |
|  | **Age group** | **Mean daily sugar sweetened soda intake (ml)** | **Mean daily sugar sweetened soda intake (12 oz serving size)** |
| **Women** | 35-44 | 128.3 | 0.36 |
|  | 45-54 | 123.4 | 0.35 |
|  | 55-64 | 66.4 | 0.19 |
|  | 65-74 | 105.1 | 0.30 |
| **Men** | 35-44 | 198.8 | 0.56 |
|  | 45-54 | 205.7 | 0.58 |
|  | 55-64 | 134.9 | 0.38 |
|  | 65-74 | 134.4 | 0.38 |
| Source: CESCAS I Study [28] | | | |

| **Table E:** comparison of two different methodologies for estimating sugar-sweetened sodas *per capita* daily consumption in Argentina | | | | | | |
| --- | --- | --- | --- | --- | --- | --- |
| **Gender** | **Age group** | **CESCAS I estimation** | | **Alternative estimation method** | | **Difference (%)** |
|  |  | **Mean daily sugar sweetened soda intake (ml)** | **Mean daily sugar sweetened soda intake (12 oz serving size)** | **Mean daily sugar sweetened soda intake (ml)** | **Mean daily sugar sweetened soda intake (12 oz serving size)** |  |
| **Women** | 35-44 | 128.3 | 0.36 | 269.6 | 0.76 | 210% |
|  | 45-54 | 123.4 | 0.35 | 259.3 | 0.73 |  |
|  | 55-64 | 66.4 | 0.19 | 139.5 | 0.39 |  |
|  | 65-74 | 105.1 | 0.30 | 220.9 | 0.62 |  |
|  | 75-84 | 105.1 | 0.30 | 220.9 | 0.62 |  |
|  | >=85 | 105.1 | 0.30 | 220.9 | 0.62 |  |
| **Men** | 35-44 | 198.8 | 0.56 | 417.8 | 1.18 |  |
|  | 45-54 | 205.7 | 0.58 | 432.3 | 1.22 |  |
|  | 55-64 | 134.9 | 0.38 | 283.6 | 0.80 |  |
|  | 65-74 | 134.4 | 0.38 | 282.5 | 0.80 |  |
|  | 75-84 | 134.4 | 0.38 | 282.5 | 0.80 |  |
|  | >=85 | 134.4 | 0.38 | 282.5 | 0.80 |  |

# References

1. Weinstein MC, Coxson PG, Williams LW, Pass TM, Stason WB, Goldman L. Forecasting coronary heart disease incidence, mortality, and cost: the Coronary Heart Disease Policy Model. Am J Public Health. 1987;77(11):1417-26. Epub 1987/11/01. PubMed PMID: 3661794; PubMed Central PMCID: PMC1647098.

2. Moran AE, Coxson P, Ferrante D, Konfino J, Mejía R, Fernandez A, et al. The Cardiovascular Disease Policy Model: Using a National Cardiovascular Disease Simulation Model to Project the Impact of National Programs to Lower Dietary Salt. In: Legetic B, Cecchini M, editors. Applying Modeling to Improve Health and Economic Policy Decisions in the Americas: The Case of Noncommunicable Diseases. Washington DC, USA: Organisation for Economic Co-operation and Development, Pan American Health Organization, World Health Organization; 2015.

3. Bibbins-Domingo K, Coxson P, Pletcher MJ, Lightwood J, Goldman L. Adolescent overweight and future adult coronary heart disease. N Engl J Med. 2007;357(23):2371-9. Epub 2007/12/07. doi: 10.1056/NEJMsa073166.

4. Instituto Nacional de Estadística y Censos. Censo nacional de población, hogares y viviendas 2010: censo del Bicentenario: resultados definitivos. Serie B Nº 2. Tomo 2 Ciudad de Buenos Aires2012 [30/06/2020]. Available from: https://[www.indec.gob.ar/ftp/cuadros/poblacion/censo2010_tomo2.pdf](http://www.indec.gob.ar/ftp/cuadros/poblacion/censo2010_tomo2.pdf).

5. Michele Gragnolati, Rafael Rofman, Ignacio Apella, Troiano S. Los años no vienen solos. Oportunidades y desafíos económicos de la transición demográfica en Argentina: World Bank; 2014.

6. Instituto Nacional de Estadística y Censos. Censo nacional de población, hogares y viviendas 2010: censo del Bicentenario: resultados definitivos. Serie B Nº 2. Tomo 1 Ciudad de Buenos Aires2012 [30/06/2020]. Available from: https://[www.indec.gob.ar/ftp/cuadros/poblacion/censo2010_tomo1.pdf](http://www.indec.gob.ar/ftp/cuadros/poblacion/censo2010_tomo1.pdf).

7. Ministerio de Salud de la Nación. Tercera Encuesta Nacional de Factores de Riesgo Para Enfermedades No Transmisibles. Presentación de los principales resultados. 2013.

8. Rubinstein AL, Irazola VE, Poggio R, Bazzano L, Calandrelli M, Lanas Zanetti FT, et al. Detection and follow-up of cardiovascular disease and risk factors in the Southern Cone of Latin America: the CESCAS I study. BMJ open. 2011;1(1):e000126. Epub 2011/10/25. doi: 10.1136/bmjopen-2011-000126. PubMed Central PMCID: PMCPmc3191438.

9. Bahit MC, Coppola ML, Riccio PM, Cipriano LE, Roth GA, Lopes RD, et al. First-Ever Stroke and Transient Ischemic Attack Incidence and 30-Day Case-Fatality Rates in a Population-Based Study in Argentina. Stroke. 2016;47(6):1640-2. Epub 2016/05/25. doi: 10.1161/strokeaha.116.013637.

10. World Health Organization. International Statistical Classification of Diseases and Related Health Problems 10th Revision [cited 2018]. Available from: <http://apps.who.int/classifications/icd10/browse/2016/en>.

11. Naghavi M, Makela S, Foreman K, O'Brien J, Pourmalek F, Lozano R. Algorithms for enhancing public health utility of national causes-of-death data. Popul Health Metr. 2010;8:9. Epub 2010/05/13. doi: 10.1186/1478-7954-8-9. PubMed Central PMCID: PMCPmc2873308.

12. Konfino J, Ferrante D, Mejia R, Coxson P, Moran A, Goldman L, et al. Impact on cardiovascular disease events of the implementation of Argentina's national tobacco control law. Tob Control. 2014;23(2):e6. Epub 2013/09/17. doi: 10.1136/tobaccocontrol-2012-050599. PubMed PMID: 23092886; PubMed Central PMCID: PMC4026283.

13. Lopez AD, Mathers CD, Ezzati M, Jamison DT, Murray CJL. Global Burden of Disease and Risk Factors. Chapter 3: The Burden of Disease and Mortality by Condition: Data, Methods, and Results for 2001. The International Bank for Reconstruction and Development, The World Bank, editors. New York: Oxford University Press; 2006.

14. García Aurelio MJ, Cohen Arazi H, Higa C, Gómez Santa María HR, Mauro VM, Fernández H, et al. Infarto agudo de miocardio con supradesnivel persistente del segmento ST: Registro multicéntrico SCAR (Síndromes Coronarios Agudos en Argentina) de la Sociedad Argentina de Cardiología. Rev Argent Cardiol. 2014;82:275-84.

15. Salgado MV, Coxson P, Konfino J, Penko J, Irazola VE, Gutierrez L, et al. Update of the cardiovascular disease policy model to predict cardiovascular events in Argentina. Medicina (B Aires). 2019;79(6):438-44. Epub 2019/12/13. PubMed PMID: 31829945.

16. Biologic Specimen and Data Repository Information Coordinating Center, National Heart Lung and Blood Institute. Framingham Heart Study-Cohort (FHS-Cohort). 2015 [30/06/2020]. Available from: https://biolincc.nhlbi.nih.gov/studies/framcohort/?q=framingham.

17. Biologic Specimen and Data Repository Information Coordinating Center, National Heart Lung and Blood Institute. Framingham Heart Study-Offspring (FHS-OS) 2009 [30/06/2020]. Available from: https://biolincc.nhlbi.nih.gov/studies/framoffspring/?q=framingham.

18. Dawber TR. The Framingham Study: The Epidemiology of Atherosclerotic Disease. Cambridge, MA: Harvard University Press; 1980.

19. Feinleib M, Kannel WB, Garrison RJ, McNamara PM, Castelli WP. The Framingham Offspring Study. Design and preliminary data. Prev Med. 1975;4(4):518-25. Epub 1975/12/01.

20. Kleinbaum DG, Klein M. Survival Analysis: A Self-Learning Text. New York: Springer; 2012. p. 366-79.

21. Biggs ML, Mukamal KJ, Luchsinger JA, Ix JH, Carnethon MR, Newman AB, et al. Association between adiposity in midlife and older age and risk of diabetes in older adults. JAMA. 2010;303(24):2504-12. Epub 2010/06/24. doi: 10.1001/jama.2010.843. PubMed Central PMCID: PMCPmc3047456.

22. D'Agostino RB, Sr., Grundy S, Sullivan LM, Wilson P. Validation of the Framingham coronary heart disease prediction scores: results of a multiple ethnic groups investigation. JAMA. 2001;286(2):180-7. Epub 2001/07/13. PubMed PMID: 24040085.

23. Liu J, Hong Y, D'Agostino RB, Sr., Wu Z, Wang W, Sun J, et al. Predictive value for the Chinese population of the Framingham CHD risk assessment tool compared with the Chinese Multi-Provincial Cohort Study. JAMA. 2004;291(21):2591-9. Epub 2004/06/03. doi: 10.1001/jama.291.21.2591.

24. Brindle P, Emberson J, Lampe F, Walker M, Whincup P, Fahey T, et al. Predictive accuracy of the Framingham coronary risk score in British men: prospective cohort study. BMJ. 2003;327(7426):1267. Epub 2003/12/04. doi: 10.1136/bmj.327.7426.1267. PubMed Central PMCID: PMCPmc286248.

25. Parish S, Collins R, Peto R, Youngman L, Barton J, Jayne K, et al. Cigarette smoking, tar yields, and non-fatal myocardial infarction: 14,000 cases and 32,000 controls in the United Kingdom. The International Studies of Infarct Survival (ISIS) Collaborators. BMJ. 1995;311(7003):471-7. Epub 1995/08/19. doi: 10.1136/bmj.311.7003.471. PubMed Central PMCID: PMCPmc2550542.

26. Law MR, Morris JK, Wald NJ. Environmental tobacco smoke exposure and ischaemic heart disease: an evaluation of the evidence. BMJ. 1997;315(7114):973-80. Epub 1997/11/20. doi: 10.1136/bmj.315.7114.973. PubMed Central PMCID: PMCPmc2127675.

27. Fernández A, Mejía RM. B.A.S.T.A. Bebidas Azucaradas, Salud y Tarifas en Argentina. Enfoque Multidisciplinario. Ciudad de Buenos Aires, Argentina: CEDES, 2018.

28. Elorriaga N, Nessier MC, Defago MD, Rubinstein A, Bardach A, Levy L, et al. Patrones de consumo de alimentos en adultos de dos localidades argentinas. In: Sociedad Argentina de Nutricion, editor. Congreso Argentino de Nutricion; Mar del Plata, Argentina. 2013.

29. Euromonitor International. Soft Drinks in Argentina. Euromonitor, 2016.

30. Drescher K, Becher H. Estimating the generalized impact fraction from case-control data. Biometrics. 1997;53(3):1170-6. Epub 1997/09/18.

31. Eide GE, Heuch I. Attributable fractions: fundamental concepts and their visualization. Stat Methods Med Res. 2001;10(3):159-93. Epub 2001/07/12. doi: 10.1177/096228020101000302.

32. Facultad de Medicina, Facultad de Economia y Negocios. Encuesta Nacional de Consumo Alimentario - Informe final. Chile: Universidad de Chile, 2010.

33. Instituto Nacional de Estadísticas de Chile. Demograficas y Vitales 2010 [cited 2018]. Available from: <http://www.ine.cl/estadisticas/demograficas-y-vitales>.

34. Ministerio de Salud. Encuesta Nacional de Salud y Nutricion 2005. Documento de Resultados 2007 [30/06/2020]. Available from: <http://www.msal.gob.ar/images/stories/bes/graficos/0000000257cnt-a08-ennys-documento-de-resultados-2007.pdf>.

35. Rubinstein AL, Irazola VE, Calandrelli M, Elorriaga N, Gutierrez L, Lanas F, et al. Multiple cardiometabolic risk factors in the Southern Cone of Latin America: a population-based study in Argentina, Chile, and Uruguay. Int J Cardiol. 2015;183:82-8. Epub 2015/02/11. doi: 10.1016/j.ijcard.2015.01.062. PubMed Central PMCID: PMCPmc4382451.

36. Ministerio de Salud de la Nación, Secretaría de Políticas Regulación e Institutos, Dirección de Estadísticas e Información de Salud. Estadísticas vitales. Información básica. Argentina ‐ Año 2015. . 2016 Contract No.: 59.

37. Stookey JD, Constant F, Gardner CD, Popkin BM. Replacing sweetened caloric beverages with drinking water is associated with lower energy intake. Obesity (Silver Spring). 2007;15(12):3013-22. Epub 2008/01/17. doi: 10.1038/oby.2007.359.

38. Imamura F, O'Connor L, Ye Z, Mursu J, Hayashino Y, Bhupathiraju SN, et al. Consumption of sugar sweetened beverages, artificially sweetened beverages, and fruit juice and incidence of type 2 diabetes: systematic review, meta-analysis, and estimation of population attributable fraction. BMJ. 2015;351:h3576. Epub 2015/07/23. doi: 10.1136/bmj.h3576. PubMed Central PMCID: PMCPmc4510779.

39. Chen L, Caballero B, Mitchell DC, Loria C, Lin PH, Champagne CM, et al. Reducing consumption of sugar-sweetened beverages is associated with reduced blood pressure: a prospective study among United States adults. Circulation. 2010;121(22):2398-406. Epub 2010/05/26. doi: 10.1161/circulationaha.109.911164. PubMed Central PMCID: PMCPmc2892032.

40. Wilsgaard T, Schirmer H, Arnesen E. Impact of body weight on blood pressure with a focus on sex differences: the Tromso Study, 1986-1995. Arch Intern Med. 2000;160(18):2847-53. Epub 2000/10/12.

41. Wilsgaard T, Arnesen E. Change in serum lipids and body mass index by age, sex, and smoking status: the Tromso study 1986-1995. Ann Epidemiol. 2004;14(4):265-73. Epub 2004/04/07. doi: 10.1016/j.annepidem.2003.08.004.
