# Supplementary material for: Projected impact of a reduction in sugar-sweetened beverage consumption on diabetes and cardiovascular disease in Argentina: A modeling study
Source: PLoS Med. 2020 Jul 28;17(7):e1003224. doi: 10.1371/journal.pmed.1003224 (PMC7386620; doi:10.1371/journal.pmed.1003224)
Supplement: S1 Protocol — (PDF) [file pmed.1003224.s004.pdf]

Research on food system policies and market innovations for

non-communicable disease prevention

Project title: Developing An Evidence Base to Inform Policymakers and other Stakeholders about Potential Policies to Reduce Sugar Sweetened Beverage Consumption in Argentina.

Short title: An Evidence Base for Policies to Reduce SSB Consumption.

Countries in which research will take place: Argentina

Funding Stream: Exploratory Research ER- 92795

Lead Applicant Organization Centro de Estudios de Estado y Sociedad –CEDES-

Mailing address: Sánchez de Bustamante 27

(C1173AAA) Buenos Aires

Argentina

Telephone number: (54 11) 4865 1070

Facsimile number: (54 11) 4865 10704

E-mail address: cedes@cedes.org

Website: www.cedes.org

Project Leader: Raul Mejia, MD, PhD

Co- Principal Investigator: Alicia Fernández, MD

Institution: Centro de Estudios de Estado y Sociedad –CEDES-

Telephone number: (54 11) 4865 1070

Facsimile number: (54 11) 4865 1704

E-mail address: raulmejia@cedes.org

Abstract

Argentina is one of the world's highest consumers of sugar-sweetened beverages (predominantly sodas) and has rapidly increasing rates of obesity and diabetes among youth and adults. Argentina has begun to implement important policies to reduce the burden of Non-Communicable Diseases. It is a regional leader in developing novel regulations on tobacco control and reduction of salt and trans-fatty acids. There is now an increasing regional public health interest in regulations on sugar-sweetened beverages to prevent Non-Communicable Diseases. As awareness of the health impact of soda consumption grows, stakeholders at various levels of government and civil society are interested in policy options to decrease consumption. In this project, a multidisciplinary group of researchers will generate rigorous evidence to inform policy development targeting sugar-sweetened beverages. It will describe the market process from its production to distribution and consumption, explore the regulations and legal framework of the market and the feasibility and impact that potential rises in taxes will have on soda consumption as well as the mechanisms that would enable these taxes to increase. By using a public health perspective, it will explore the magnitude of cardiovascular morbidity and mortality benefit associated with the proposed fiscal policies. The project will determine health and health cost benefits of a reduction in soda

intake associated with possible price increases over the next decade. Women stand to bear a disproportionate burden of the predicted global increase in non-communicable diseases. Thus, all analysis will consider possible differential impacts of policies by socioeconomic status and gender. The project team includes physicians, lawyers, sociologists, and economists, and includes expertise from the US and Mexico. Knowledge translation will include a wide dissemination of results and the development of partnerships with health advocates, decision makers and other local and international players, such as the Pan American Health Organization.

Research Background, Focus, Problem Statement and Justification

Non-communicable chronic diseases (NCDs) are increasingly common in Latin America and throughout the developing world. The prevalence of modifiable NCD risk factors – obesity, lack of exercise, tobacco and unhealthy alcohol—increases with urbanization, poverty, and social inequities. Without appropriate action, these factors will contribute to an enormous increase in the burden of disease in Latin America.

Sugar sweetened beverage (SSB) consumption is closely associated with obesity and diabetes prevalence. Multiple studies have shown that SSB consumption leads to obesity(1) (2, 3), and to diabetes(1, 4). Cardiovascular disease is strongly associated with SSB consumption not only via the development of diabetes but also through increases in weight and hypertension(5-7, 8{Malik, 2006 #192)).

A recent report, "Ultra-processed food and drink products in Latin America: Trends, impact on obesity, policy implications, (9) found that per capita sales of these products increased by 48% in Latin America from 2000 to 2013, even as such sales were declining in North America. These trends stem from changes in the international food system brought about by globalization and market deregulation, which have increased the penetration of foreign and multinational food corporations into national markets. Increased Latin American SSB consumption during this time period was strongly correlated with local body weight increase, suggesting that these products are a major driver of growing rates of overweight and obesity in the region.

To slow the rise in consumption of ultra-processed foods and increasing rates of obesity and overweight in Latin America, the WHO and PAHO recommend that governments, the scientific community, and civil society organizations support and implement policies to protect and promote healthy food choices(10). These include consumer information and education campaigns and also broadly directed policies, such as regulations on pricing, incentives, agriculture and trade. These measures are in line with PAHO/WHO's 2014 Plan of Action for the Prevention of Obesity in Children and Adolescents, which also calls for strict limits on marketing of unhealthy food products to children (10).

Several countries have considered or have implemented restrictions or fiscal regulations (taxes) on sodas. In the US, for example, it has been demonstrated that adding an excise tax to sodas could dramatically reduce medical costs due to cardiovascular diseases and significantly increase tax revenues(11-13). Mexico has been working over the last 10 years to control obesity and to reduce SSB intake. Based on locally produced, solid scientific evidence, Mexico implemented an excise tax on sodas and passed legislation to prohibit selling SSBs in schools (14, 15). A growing body of research supports the Mexican strategies as effective(16).

Cardiovascular diseases (CVD) are the leading cause of death in Argentina and are responsible for 34% of total deaths(17). Obesity and diabetes are growing particularly rapidly in Argentina. The National Risk Factor Survey (ENFR) reported an increase in obesity/overweight rate from 49% of adults in 2005 to 58% in 2013, with 21% being obese(18). Diabetes prevalence has increased as well, from 8.4% in 2005 to 9.8% in 2013(18). The Argentine Ministry of Health (MOH) projects that the prevalence of adult diabetes will be 11.7% in two years (19). Both obesity and diabetes are more common among the poor (20), and many people with diabetes remain undiagnosed and are not included in these numbers(21). Among adolescents, overweight and obesity are quite prevalent at 19% and 2,6% respectively.

Argentina is among highest consumer of sodas in the world, and perhaps even the highest. (http://www.npr.org/sections/goatsandsoda/2015/05/20/408027045/id-like-to-buy-the-emerging-world-a-coke); Argentina is the highest consumer of sodas in the world, on average per capita consumption is 131 liters per year per habitant(22). Consumption of SSB rose 218% in the last 30 years, from 1352 million liters per year in 1982 to 4304 million liters SSB per year in 2012 (Figure 1). Use is common at every level of Argentine society, (including among high SES families) and in multiple settings, including private and public schools. More than 50% of the population have lunch or dinner with sodas, and a WHO survey found that 66% of Argentine adolescents ages 13-15 consume soft drinks one or more times a day (23) (24). In Argentina the soft

drinks market is led by regular sodas; Coca-Cola and Pepsico produces nearly 90% of local production of soft drinks and in economic terms this represents more than $ 8,500 million (USD 1,000 millions) per year (23)

Trend in SSB Consumption per year in Argentina (million liters)

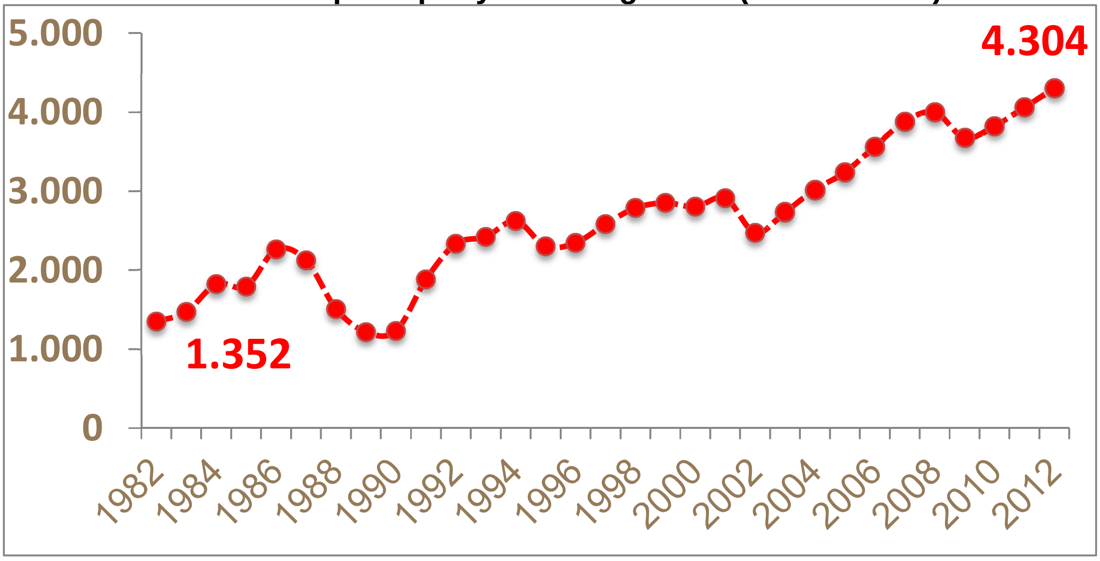

National Congress 2014

In the past decade Argentina has implemented important policies at the national level to reduce the burden of NCD. The National Law for Tobacco Control, the national law that reduces significantly the content of salt in processed food, and the national law that prohibits almost all trans-fatty acids in processed food are examples of successful policy implementation. However, very few efforts have been made to reduce SSB consumption. There is a current policy window, as the Ministry of Health is interested in enhancing food regulations to improve health. Argentine policymakers and stakeholders from civil society need rigorous research on SSB policy options to propel discussion and inform options.

To our knowledge, no Argentine or international research group is working on generating evidence surrounding the health effects of SSB in Argentina, or examining the local fiscal and regulatory interventions that could be implemented to decrease SSB consumption. In this project, we will use a rigorous multidisciplinary approach to provide a corpus of evidence-based documents useful to policy makers and other stakeholders about the fiscal and regulatory interventions that could be implemented to decrease the consumption of sodas We will also estimate the health and economic impacts of these potential policies. This information will be generated from a combination of international and Argentine specific evidence and could be used by other countries of the region interested in tackling the consumption of sodas.

Contribution to IDRC’s Strategic Objectives

This proposal will contribute to IDRC´s strategic objectives in different ways

Strategic Objective 1. *"Invest in knowledge and innovation for large-scale positive change"*

This exploratory project will establish a solid evidence base for the development of targeted and strategic policy initiatives aimed at reducing SSB consumption. The ultimate target of the project is large scale positive change, that is to say, the successful implementation of effective SSB policies. The project is highly innovative in that is generates new knowledge for Argentina using multiple approaches, including a human rights-based legal framework which has not been previously used for SSB policies. Finally, the project's approach is to involve key stakeholders at all stages so as to provide valuable input for policy development, result dissemination, and advocacy.

Strategic Objective 2. *"Build the leaders for today and tomorrow"*

This multidisciplinary project will engage investigators and consultants from the Ministry of Agriculture, the Ministry of Health, the Universidad de Palermo, Argentina, the National Public Institute of Mexico INSP, the University of California San Francisco and from CEDES. CEDES has a strong history of cooperation with local non-governmental organization and with the Ministry of Health, and provided key scientific evidence to support the implementation of policies for tobacco control(25), salt reduction(19, 26, 27),and trans fatty acids reduction(28). A recent study supports the provision of lipid lowering medications at no cost to the underserved in the national health program. (Konfino J, Fernandez, A, Mejia R, et al in press). The multidisciplinary nature of the proposed project that spans multiple institutions and diverse disciplines will strengthen institutional ties, develop an innovative health policy research group capable of incorporating and mentoring young leaders in research, and increase health policy research capacity in Argentina.

This project has intentionally integrated young investigators who demonstrate exceptional promise as knowledge leaders in the field of NCD. Project Principal Investigators have extensive experience in mentorship and a strong commitment to mentoring junior investigators. Dr. Alicia Fernandez is the recipient of multiple awards for mentorship from UCSF and US national institutions (SGIM, The Arnold P. Gold Foundation.) She was recently awarded a Midcareer Investigator Award in Patient-Oriented Research (K24**)** from the US NIDDK/NIH and is lead mentor on multiple NIH grants. Dr. Raul Mejia was recognized in 2008 with the Global Health Leadership Award from the Global Health Research Initiative, Canada for his mentorship in the *Argentine Tobacco Control Research Mentorship Program.* His efforts enabled the rise to leadership of several junior researchers within their own institutions, and in the Ministry of Health(29).

The PIs will serve as mentors for the younger researchers of the team including Maria Victoria Salgado, Sonia Ariza, Cecilia Garboti, Jonatan Konfino, Lorena Peña y Tamar Finzi. Drs. Maria Victoria Salgado and Jonatan Konfino will conduct the health consequences analysis with the CHD Policy Model CHD Policy Model; Alicia Fernandez will be their principal mentor with Raul Mejia's assistance. Tamar Finzi and Lorena Peña, will develop the Stakeholder´s mapping and the Force Field analysis; Raul Mejia will serve as their lead mentor. Paola Bergallo will mentor Cecilia Gariboti and Sonia

Ariza for the analysis of current legislation. The ultimate aim of the mentoring component of this proposal is to develop these researchers as local leaders in policy oriented research on NCD.

Strategic Objective 3. *"Be the partner of choice for greater impact"*

We have two goals within this project that will strengthen partnerships and multiply resources. First, is to complete the Stakeholder Influence Map that will identify stakeholders interested in reducing SSB consumption (such as child health advocates) as well as those interested in promoting sugar production and consumption. The second is to establish partnerships with organizations interested in reducing SSB consumption and work with them to establish mutually agreed upon ways of disseminating key information, such as a Sugar Science Website and workshops. We will be assisted in this process by Ms. Sarah Fine, a consultant with extensive expertise in coalition building and policy advocacy in Argentina.

We will also leverage this project to strengthen international collaborations re obesity prevention efforts. For example, we have had early conversations with officers from Bloomberg Philanthropies who have expressed interest in collaborating with this project, and with representatives of FIC Argentina (with whom we have worked in the past on projects aimed at reducing salt and TFAs in processed food). We plan to continue to pursue those partnerships.

Finally, we will work with investigators and advocates from the INSP, Mexico who will provide us with the collaboration of INFORMAS (International Network for Food and Obesity/non-communicable diseases Research, Monitoring and Action Support) so as to capitalize on the benefits of international collaboration and facilitate the dissemination of the project results at a regional level.

Objectives of the Project

General Objective

The overall goal of this research is to provide relevant evidence base to inform policy makers and other stakeholders in Argentina when considering policies to reduce consumption of sugar-sweetened beverages, and specifically evidence associated with a possible tax on these products.

Specific Aims

1. To develop a context map of sugar-sweetened beverage production, distribution and consumption in order to assess the feasibility of a rise in SSB tax;

2. To analyze the national legal and regulatory framework for the production, distribution and sale of sugar-sweetened beverages;

3. To describe the impact of tax and subsidy schemes on the sugar-sweetened beverage value chain, from the sugar cane sector down to consumer sales;

4. To describe the price elasticity of sugar-sweetened beverages amongst different populations and policy intervention scenarios

5. To model the impact of changes to sugar-sweetened beverage consumption on population cardiovascular health and related health-care costs

6. To inform policy options and fiscal taxation alternatives for sugar-sweetened beverages in order to contribute to the prevention of overweight, obesity and diabetes in Argentina.

Methodology and methods

Research on SSB requires the involvement of multiple disciplines and the engagement of diverse stakeholders in order to address the complex implementation challenges they face. This project will include qualitative instruments, legal analysis, econometric studies and computer simulation models to generate a corpus of evidence from diverse perspectives that will be assembled and discussed with key stakeholders. The project aim is to identify actors from relevant sectors (government, civil society, and private sectors) and explore how they can be engaged in the design and implementations of policies for reducing obesity in Argentina. The final result will be a Policy Influence Map that will be able to be implemented in the short term. Policy Influence Maps are a useful way to demonstrate the relative size of key actors, interactions among them, and key policy levers. Constructing a useful and broadly accepted Policy Influence Map will allow stakeholders to debate and agree upon key levers and will generate dialogue useful to collaborative action.

Because women stand to bear a disproportionate burden of the consequences of the twenty-first century's predicted global increase in non-communicable disease (NCD) prevalence and are particularly susceptible to the harms of nutrition transition (10), we plan to perform all the analysis of legislation and the economic analysis using a gender perspective. CEDES has a long history in research projects based on gender inequalities, As is depicted in their curriculum vitaes Raul Mejia, Paola Bergallo and Tamar Finzi have published several studies of gender issues in different areas of public health including reproductive health, women´s rights, tobacco consumption, among others

Finally, environmental sustainability will be considered in the discussion of policies to reduce SSB consumption by including preservation of fresh water and the final disposal of plastic recipients**Specific Aim 1:** *To develop a context map of the setting that surrounds SSB production, distribution and consumption.*

The focus of this aim is to analyze and describe the production, commercialization and consumption of SSB, as well as to examine the relevant political context of Argentina in order to explore SSB policies that could be instituted. We have identified the following sub aims.

:

Specific aim 1.1: To describe the sugar industry in Argentina over the last 10 years. To fulfill this objective, we will analyze variables related to production, commercialization

and consumption. We will consider environmental impact and public health consequences as cross cutting issues. We will identify the most significant regions of sugar and SSB production in the country, looking upon the dimension of the gross value, exports and investments made in the last decade, profitability and activity costs.

First, we perform a comprehensive review of the literature focused on specialized publications of the agro industry and food industry, such as Énfasis and Agropost. This review will include websites related with sugar, sugar cane and SSB such as: www.alimentosargentinos.gob.ar; https://www.wto.org/indexsp.htm, http://www.industriaalimenticia.com, www.cepal.com, http://www.inv.gov.ar/ and http://www.usda.gov/wps/portal/usda/usdahome?navid=EN_ESPANOL.

We will search for newspaper articles that contain data and information on the production, commercialization and consumption of SSB, not only on a national and provincial level (Salta, Tucuman, San Juan, Cordoba) but also in other neighboring countries like Bolivia, Brazil, Chile, Mexico, Peru and Uruguay.

The available data from national statistics will also be reviewed and taken into account, mainly the Argentina Industrial Product Statistics (Estadísticas de Productos Industriales, EPI) and studies of supermarket sales, especially the section about supermarket business.

After completion of the review we will conduct at least 12 in-depth interviews with key stakeholders involved in the areas of production and commercialization of SSB. We will use a “snowball strategy” to enhance the sample of stakeholders. This is a preliminary sample estimate because this will be a constructivist inquiry and our preliminary theory would be expanded or modified in the context of the study. Looking for data that will support or challenge our understanding we will interview informants until the “saturation criteria” is reached[44] Two different guides for these interviews will be designed, one for stakeholders from the production of sugar and other for stakeholders related with manufacturing and commercialization. Some examples of key informants that might be invited to participate are the Centro Azucarero Argentino (Argentinian Sugar Center) and Distributor Reginald Lee (distributor Coca-Cola)

Specific aim 1. 2: Design and produce a stakeholders map describing the actors involved in the production, commercialization and consumption chain. We will interview these stakeholders in order to precisely describe the path from sugar cane to SSB household consumption. Key elements of each interview include a) description of the sector, i.e. number of workers, factories, businesses involved b) key regulations that shape business practices c) key elements of taxation relief or taxation incurred in their sector, d) perspective on additional consumption taxes; e) key sector arguments against consumption taxes. The information gained from these interviews will be invaluable in mapping key stakeholders and understanding policy options and policy argument in favor and against regulatory and tax-based approached.. This information will be used to construct a Policy Network Map, a Stakeholder Influence Map and to complete a field-force analysis.

All relevant stakeholders will be identified and classified into primary and secondary actors, according to their relevance to SSB production and consumption and their influence over market regulations. Then, the relationships between stakeholders,

potential conflicts of interest, and the appropriate level of stakeholder participation in SSB production, sales and consumption will be assessed.

The technique employed for the collection of data to fulfill this objective will be at least 18 in-depth interviews to different stakeholders involved in SSB. An interview guide will be designed and piloted within the research group. The final list of interviews will be built jointly with the lawyers and the economist of the project to ensure that the information obtained could be used for all the objectives addressed in the proposal. Some examples of key informants are; Subsecretaría de Agregado de Valor y Nuevas Tecnologías (Sub-secretariat for Added Value and New Technologies) and Centro de Estudios sobre Obesidad Infantil (CESNI)

Specific aim 1. 3: Develop an advocacy plan to reduce the consumption of SSB in Argentina. To achieve this, first a force-field analysis technique will be employed with the main goal of identifying the pressures for and against changes in current SSB government policies. The work methodology for the gathering of this information will be a stakeholder workshop in which the actors identified in “Objective 2” will participate as well as expert consultants involved in the project such as Amalie Ablin from the Ministry of Agriculture. Prior to the workshop an online-survey will be administered to its participants in order to weight their opinions related to different aspects of the SSB consumption and a potential tax on them and its possible externalities. After the workshop, an advocacy plan will be developed by all the participants of the project included the consultants Simon Barquera, Sarah Fine and Amalie Ablin. This draft advocacy plan will be further refined with stakeholder partner input.

Specific aim 2. *To complete a situation analysis of the Argentine legal and regulatory framework for the production, distribution, and consumption of SSBs under a human rights framework.*

As WHO, governments, and scholars have come to recognize recently, formal and informal regulations, a human rights based approach, and revitalized accountability mechanisms are major tools in shifting norms towards more nutritious and healthy lifestyle habits(30-32). Assuming the centrality of legal regulations, this component of the research seeks to produce a situation analysis of the Argentine and comparative legal framework regarding SSBs in order to contribute to combat obesogenic environments across the country.

This subproject is divided into three parts aimed at producing the legal data necessary to evaluate current SSBs’ regulations and to assess, ground, and design potential reforms and accountability mechanisms for Argentina. With that goal in mind, the first part will describe current applicable laws, reform bills and accountability forums; the second part will offer a comparative survey of recent SSBs’ regulatory and accountability experiences in other countries; and, the third part will provide the human rights grounds for potential legal SSBs restrictive reforms.

This component of the project seeks to answer the following questions: (a) what are the current laws and regulations applicable to SSBs at the international, the national and the sub-national levels?, (b) what accountability forums and tools are currently available to monitor SSBs regulations, (c) what is the legal and empirical evidence available on concrete episodes of SSBs regulations adopted in other countries?, (d) what barriers

have been faced in the enactment and implementation of such regulatory initiatives and what were the arguments grounding for and against them, (e) how can comparative SSBs' regulatory episodes inform the development of reform proposals for Argentine law, (d) how are SSBs regulations legally justified?, more specifically, how do SSBs regulations intersect with constitutional and human rights framework?, and what arguments are more suitable to ground prospective SSBs regulations and to answer opposition strategies?

From a methodological standpoint, the first part of the research is descriptive and seeks to offer a systematization of the body of rules, precedents and accountability settings scattered through diverse policy areas and at different levels of the governmental structure and a variety of jurisdictions. The second part of the project will adopt a descriptive, comparative, and socio-legal approach in order to map out recent regulatory initiatives and to contrast impacts and implementation barriers from the perspective of both the laws "in the books" and "in action." Finally, the third section's stance is conceptual and justificatory and will systematize and develop pro-SSB's regulatory moral and legal arguments.

The first part of the legal component of the project will produce a comprehensive review of the international, national and provincial legal regulations and judicial precedents that rule the production, distribution (including inter-jurisdictional commerce), and consumption of SSBs in the country. More specifically, we will survey and systematize domestic laws regulating the sugar market; SSBs' composition, promotion, and labeling conditions; obesity laws; healthy food supply in schools and other settings; the tax system; and SSBs' rules on availability in communities and stores. We will look both at formal laws and soft-law initiatives such as nutrition guidelines, codes of conduct, conflicts of interest regulations, or professional recommendations. We will also identify SSBs industry's self-regulatory initiatives such as those limiting the appearance of children in SSBs advertisement. Finally, following Swinburn et al.(32), the research will also map out existing mechanisms to monitor compliance, investigate complaints, and enforce SSBs regulations, such as agencies, auditors inspectors, or commissioners. Moreover, it will also survey accountability tools to assess procurement contracts and grant requirements for government agreements of food supplies (i.e. hospitals, schools, public agencies), and litigation against the SSBs industry.

The second part of this component will survey comparative regulatory experiences from a legal perspective. We expect to identify the different policy regulation alternatives that have been successfully applied as well as contentious experiences in other countries as a starting point to consider the advisability to adapt such regulations to Argentina. As in other comparative observations(33), in our study of a selected number of comparative legal regulations we will examine the legal bases for and against the development and implementation of such rules, the accountability mechanisms set up to monitor compliance, the level of government at which laws were implemented, the barriers to using law faced, and the role of key actors such as the public health community or the SSBs industry. The selection of comparative regulatory episodes will include at least one experience where strategies have adopted a gender perspective, sensitive to women's role both as SBBs' consumers and as main caregivers, and hence, responsible for children's health and nutrition education. Ultimately, this survey of comparative law

sources and legal reform cases will provide key information to understand the regulatory models and stages of prospective legal reforms in the Argentine context.

The third part of this component will survey the constitutional and human rights arguments justifying and opposing a selection of regulatory strategies aimed at effective SSBs' reduction policies. We will systematize and develop pro-regulatory arguments based on the right to health and other human rights contemplated in the Argentine Constitution and human rights treaties incorporated to it. Moreover, we will put a special focus on gender equality and other women's rights arguments regarding gender based discrimination and justifying regulatory interventions targeting women. This is a key aspect if we understand women both as human rights holders and as providers of health and care within their communities.

Finally, adopting a human rights approach we will discuss and respond to business-protective arguments, such as the right to free trade and association. For this part of the component, we will survey and analyze relevant human rights precedents from domestic judicial systems and international human rights law.

The three parts of this component will supply necessary inputs to legally ground and to model the regulation of prospective policy changes and legal advocacy initiatives. We will produce legal information that has not been systematized for Argentina taking into account its complex federal structure as well as the important existing relationship between constitutional norms and international human rights law since the 1994 constitutional amendment.

Specific Aim 3: *Analysis of Taxation*

The field of health programs and systems research recognizes many alternative ways to affect the consumption of SSB: communication campaigns, promoting healthy habits through family doctors and broad policies such as defining the set of foods and beverages that might be included into a subsidized basket for the poor.

From the economic perspective, the use of tax and subsidies schemes along the chain of production and consumption of SSB can be seen as an accurate tool to support these types of interventions. . In all cases, the goal is probably similar: align private behavioral patterns with those of the society, considering the chain of benefits and costs related to a deterministic relationship between SSB consumption, obesity and cardiac illness, which affect life expectancy and quality, as well as health expenditures.

The goal of this component of the proposal is to identify and to analyze current taxes in Argentina which directly or indirectly affect the production and consumption of SSB, and to discuss their potential effects on this specific sector. Specifically, we propose to review progressive and regressive tax structures implemented in the country (income and property direct taxes, as well as indirect taxes on consumption, among other specific fiscal tools) in order to understand the scenarios triggered by each of them, the goals intended to pursue, the way these selected interventions were implemented, and the results obtained, if they were documented.

The current tax structure will be discussed within an historical perspective, considering a fifteen-year scenario, back to the beginning of the century. In addition, the findings will

be analyzed under the light provided by the evidence of similar policies and results obtained from other experiences in the Latin American region and OECD nations.

The analysis of tax intervention patterns in the country will be introduced by the discussion from the economic literature about how potential economic interventions in the vertical scheme of SSB production generate incentives and redefine conducts in consumers and producers. Based on the general framework provided by the New Theory of Regulation under uncertainty(34) the analysis of taxation requires anunderstanding of how vertical and horizontal contractual structures under the industrial organization of the SSB industry are affected by different fiscal policies along the chain of production. Specifically, different structures in property rights may provide alternative reactions to similar policies, based on the characteristics of competitiveness in each production stage –sugar, beverages, wholesale distribution, retail-, the level of diversification of the production function, as well as the current mechanisms of commercialization of their goods.

Specifically, , this component will benefit from the stakeholder analysis at the industry level developed in specific aim 1. It will contribute by incorporating an economic policy making perspective, helping to create a comprehensive and enriched taxonomy of the different types of tax interventions, and how different tax designs affect the relationships within the SSB sector, from the production of inputs until the retail price of beverages and their impact on commercialization.

The final section of this component will contrast the studied regulatory interventions with the evolution of SSB prices and units sold in the market, providing inputs for a systemic approach towards the design of a consistent public policy. The specific goal of this analysis is to search for potential correlations between tax policies affecting the SSB industry in the country, with the production/consumption patterns shown by the SSB supply side as well as from household surveys on family consumption during the period under study.

The information will be obtained from different chambers of producers along the SSB system, and Surveys of Supermarkets, comparing a span of fifteen years. The information will be adjusted at constant prices by using price indexes provided by INDEC (the National Statistics and Censuses Institute), as well as from other private institutions providing information on prices.

Finally, these indicators will be complemented with information on household surveys, extracting information adding to information about the distribution of family consumption across groups of goods and services, with the use of other food and beverages as control variables. We will be able to provide data about the weight of beverages by household sensitive characteristics, such as presence of children, women head of family, head of family's level of education, among others.

Specific Aim 4*: Economic Analysis of Consumption*

Analyzing the structure of taxes in the SSB market is a necessary step in order to identify the potential impact of monetary measures targeting SSB consumption. The challenge of measuring such impact rests in the fact that people's preferences on SSB consumption are unknown, both by policymakers and the researchers. Making this more

complex is that those preferences are not homogeneous across groups, likely varying by income, household composition, sex and age. Particularly, in markets like SSB, products on sale are not homogeneous, based on brand characteristics, flavors, etc. The presence of heterogeneity allows the choice of products within a relatively high variety, where selection not always is associated with lower price, but with fads and patterns triggered by communication campaigns, loyalty to the brand, etc.

The mechanism of modeling consumers' behavior takes the form of discrete choice models of product differentiation, where alternative patterns of demand are tested in order to identify the "true model" that allow the prediction of consumption. Once such demand structure is estimated, the effects of taxes on consumption are able to be calculated.

An original specification introduced by McFadden(35) considers the estimation of a demand function where the probability of buying a specific product "i" relies on its price and its product characteristics, following the structure of a decision tree. Later on, Berry(36) proposes a specification to estimate consumers' choices under uncertainty, departing from an individual utility function:

$$U_{ij} = f(\varsigma_i, p_j, x_j, \xi_j; \theta)$$

Where individual i chooses to buy product j based on personal characteristics, product price, non-price product characteristics, and un-observable product characteristics. The model intends to calculate which is the vector of coefficients for each explanatory variable. Each consumer i decides to buy product j if it is preferred to any other product r:

$$U_{ij}(\varsigma_i, p_j, x_j, \xi_j; \theta) \geq U_{ir}(\varsigma_i, p_r, x_r, \xi_r; \theta)$$

Under a general specification of the utility function as follows

$$U_{ij} = f(\varsigma_i, p_j, x_j, \xi_j; \theta) = x_j.\beta - \alpha.(p_{fj} + p_{vj}.y_i) + \xi_j + \varepsilon_{ij}$$

a cumulative utility function for all consumers takes the form:

$$F(\varepsilon_{ij} < \varepsilon) = e^{-e^{\varepsilon_{ij}}}$$

it follows that the participation (s) of each good in the market j is:

$$s_j(\delta) = \frac{e^{\delta_j}}{(\sum_{r=0}^{N} e^{\delta_r})}$$

Taking natural logarithms in both sides of the equation, the final function to estimate is:

$$Ln(s_j) - Ln(s_0) = \delta_j = x_j.\beta - \alpha.p_j + \xi_j$$

From where the own and cross demand elasticities n (with other alternative products) are, respectively:

$$\eta_{jkt} = \begin{cases} -\alpha . p_{jt} . (1 - s_{jt}) \\ \alpha . p_{kt} . s_{jt} \end{cases}$$

In our particular case, and in order to calculate the behavioral responses triggered by such policy, we will use alternative estimation models of demand, providing information about the intensity of response of potential interventions affecting sales prices. Traditional estimation methodologies will be implemented, following log specifications, SEDS (substitution elasticities demand systems), as well as AIDS (almost ideal demand systems) and QUADAIDS (quadratic AIDS) models, used to estimate Engel's curves. Comparative analysis will be developed in order to provide robust results, and enhancing the simulation of policy implementation.

In every case, the estimation departs from cumulative individual utility functions defining total sales by type of beverages, and looks for identifying coefficients that may be used to calculate own and cross price demand elasticities across SSBs. In turn, these elasticities will be applied to create alternative tax scenarios, providing an array of sensitivity analysis to the implemented policy.

The research team also proposes to estimate potential differences in elasticities across income groups, regional settings, households' educational patterns, and family composition (i.e. below-14-year-old children), under the assumption that socioeconomic characteristics are relevant to identify and implement tax policies. Particular interest is defined by the role played by women when they are considered head of household, because of the impact that generally has on family consumption patterns in health and purchase of goods and services. The econometric implementation, therefore, will add these vectors of variables to the traditional estimation setting, aimed to improve the policy analysis and the structure of. In addition, SSB consumption is surely affected by changes in the supply of new alternative drinks (light and flavored beverages), which were brought to the market during the last years, and need to be included in the current scenario.

The empirical implementation will take advantage of a relatively wide range of available information, particularly National Household Expenditure Surveys (ENGH, in Spanish) in 1997/8, as well as 2004/5 and 2012/13, which provides eighteen sets of foods and beverages consumed by Argentine families in a by-region representative sample of about 26,000 observations each. Additional information to identify price evolution by type of SSB will be obtained from the Supermarket Surveys as well as the Argentine Chamber of SSB Producers and Euromonitor.

Specific Aim 5: *Modeling the Impact of SSB consumption changes on Health Benefits and Health Care Costs*

Although clinical trials are still considered to be the gold standard for best practice evidence for cardiovascular prevention, their applicability to assess health intervention effects on entire populations has been questioned. Computer simulation models can assist with this problem, by scaling up the evidence to a broader, more diverse population, and because their ability to estimate intervention effects over different periods of time. They can also be used to compare different subpopulation, or to

compare effects among the same group of people when applying different interventions(37).

The Cardiovascular Disease (CVD) Policy Model is a computer simulation, state transition (Markov cohort) model that estimates the prevalence and incidence of cardiovascular disease, as well as its related mortality and direct associated costs, by using epidemiological data of the population 35 years old and older. Every adult alive in a given simulation year must be in a healthy or diseased state, and their risk is distinctively calculated according to their age and gender. Each simulation is run on annual basis, and outcomes change over time according to population's census projections(38, 39).

The CHD Policy Model will be used to provide a body of evidence on the impact of SSB consumption on cardiovascular health, which in term will support health policy makers and other stakeholders in Argentina considering measures to lessen SSB consumption.

Summary of steps

1. To update the Argentinean version of the CVD Policy Model.

2. To review the literature in order to estimate the effect of SSB consumption on body mass index (BMI) and obesity, diabetes and hypertension.

3. To estimate the average SSB serving size in Argentina

4. To examine and project the health and health care cost benefits of a reduction in SSBs intake associate with possible price increases over the next decade, using the Argentina's version of the Cardiovascular Disease Policy Model.

Update of Argentina's CVD Policy Model

Although originally design for the US population, the CVD Policy Model can be used by any country if sufficient input data is available(40). The current Argentina version of this model was developed in collaboration with researchers from Argentina's National Ministry of Health and from University of Buenos Aires(25). Since it was last updated, new local data sources have become available. Among others, the annual health and health cost statistics reports from The Ministry of Health, the national census conducted in 2010, the 2013 National Risk Factor Survey, and the CESCAS I study, an on-going observational prospective cohort designed to study cardiovascular disease prevalence and risk factors in the Southern Latin America(41). The model's input files will be modified with the information provided by these sources, updating its data on population projections by age and gender, prevalence and incidence of CVD events and risk factors, and direct health cost estimations. The data is therefore included in the input files of the CVD Model.

Model Calibration

Once an updated version of the model is developed, a baseline simulation (that is, a simulation of the population CVD outcomes assuming no intervention at all) will be run. The model predicted number of deaths per year will be compared to health statistics in Argentina. The actual total number of deaths attributable to coronary heart disease will

be estimated as a compound of both definite CHD deaths in health records plus a percentage of poorly defined deaths (named ‘garbage’ codes, already defined) that could be attributed to CHD deaths}. When finished, we will count with a most recent version of the Argentinean model.

CVD Policy Model and Sugar Sweetened Beverages

SSBs are one of the main sources of calories in the western diet, and SSB consumption has been linked to changes in Body Mass Index (BMI), diabetes, and hypertension (HTN)(8, 42). In the CVD Policy Model, both diabetes and hypertension are directly linked to a greater risk for CVD events, and diabetes is also linked to non-CVD mortality. Then, it will then be necessary to estimate how SSB affect CVD risk factors and outcomes, the actual inputs of the model.

In a recent meta-analysis, one serving/day of SSB was associated with a 13% increase incidence of type 2 diabetes, after adjusting for adiposity(43)}. The same meta-analysis estimated that, over 10 years, SSB consumption would be responsible of 1.8 million events of type 2 diabetes in the US and 79000 in the UK, which represents a population attributable fraction of 8.7% and 3.6% respectively.

In addition to the direct effect on diabetes and HTN, SSB also have an effect on CVD outcomes that is mediated through changes on BMI; SSB consumption has been associated with higher BMI in multiple studies(8, 13, 42). Nevertheless, the effect of a reduction of SSB consumption on BMI at a population level is less clear; the extent of calorie substitution following a decrease in SSB consumption is difficult to assess beforehand. Using a similar model to the one applied in a California’s SSB study(13), and based on the calculation of 3500 kcal/pound, we will run 3 different scenarios (simulations), while retaining the independent effects on diabetes and blood pressure:

1) Scenario 1: it will be assumed that the entire impact of a decrease in calories due to a reduction in SSB consumption would be translated to weight loss

2) Scenario 2: the simulation will assume that 39% of the calories will be replaced by other sources of calories, and the net calorie reduction translated to weight loss will be 61%

3) Scenario 3: A reduction in SSB consumption has no impact on body weight, assuming a complete substitution with other sources of calories

Moreover, weight loss is a dynamic process that depends on factors besides the calorie intake, such as the adiposity(44). Therefore, and based on the model propose by Hall et al, we will then run a sensitivity analysis to compare if the CVD outcomes assuming a lineal relationship between weight loss and reduction of calorie intake changes significantly when assuming a dynamic weight loss process(44).

The last requirement to be able to run simulations of SSB consumption reductions will be to determine the average serving size of SSB in Argentina. Previous data estimated the annual consumption of sugary soft drinks in Argentina to be near to 140 liters per capita. More updated information provided by the Ministry of Agriculture will be used to calculate the average serving size of SSB.

Then, the CHD policy model will be used to examine and project the health and health care cost benefits of a reduction in SSB intake associated with possible price increases over the 2018-2027 period. The economic team will estimate the price-demand elasticity to calculate the level of tax change required to obtain different reductions on SSB consumption. Based on these results, we will run simulation models that will estimate the preventable cases of incident diabetes and coronary heart disease, as well as all-cause mortality and health related cost reductions, if a drop of 5% 10%, 20% or 50% in consumption of SSB were to be achieved.

Specific aim 6: *To inform policy options and fiscal taxation alternatives*

Despite that the objective of this exploratory project is to get information from different actors and diverse perspectives in order to develop a Policy Influence Plan. We will implement a Knowledge Exchange Strategy to raise awareness among stakeholders about the Public Health consequences of SSB consumption and possible alternatives to decrease consumption. We will conduct ai workshop with different stakeholders to discuss the results of the project and the best way to propose and implement an increase in SSB taxes. This workshop will start a serine of Policy Dialogues to be implemented in the short term

Brief description of the policy and private sector landscape

The Argentine Ministry of Health is addressing NCD prevalence through the *Plan Argentina Saludable* which focuses on promoting the reduction of the salt and trans-fatty acids content in processed foods and encouraging the provision of healthy diets in public school(45).Based on robust local research results(19) the MoH has implemented a National Law for Tobacco Control, a National law to reduce Salt content in processed food, and a National Law to reduce Trans Fatty Acids content in food but has not yet implemented any action to reduce SSB intake. Discussion on inclusion of SSBs issues in the *Plan Argentina Saludable* (including SSB taxes and policies directed at limiting consumption of SSBs is schools) has begun at the MOH. Recently a bill was submitted in the National Congress to increase the price of SSB by $0.45 (USD 0.045) for the purpose of creating an athletic center. The lack of an evidence base of possible options and their projected impact and the lobby campaign implemented by SSB producers has hindered the debate.

In response to recent news media reports on the danger to public health associated with SSB soft drink manufacturers have mounted an aggressive advertising campaign to discredit the information. The campaign is based mainly on denying the contribution of SSB calories the local diet, emphasizing that beverage consumption is a personal choice, and promoting corporate social responsibility campaigns. This is similar to what has been seen in other countries and we anticipate that's just the beginning of an aggressive strategy to avoid regulations and the “denormalization” of SSBs consumption.

Expected research results and intended outcomes

During the project, the researchers will interact with representatives of the Ministries of Health, Economy and Agriculture as well as civil society organizations such as the Interamerican Heart Foundation (FIC Argentina), the Centro de Estudios sobre Nutrición Infantil (CESNI), representatives of the National Congress among others. In addition Daniel Ferrante is an officer of the Ministry of Health and Amalie Ablin works at the Ministry of Agriculture. Despite that the objective of this exploratory project is to get information from different actors and diverse perspectives in order to develop a Policy Influence Plan to be implemented in a following phase of the project, we expect that conducting this research will raise awareness among stakeholders about the Public Health consequences of SSB consumption and possible alternatives to decrease consumption. We will invite representatives of all these sectors to a workshop to discuss the results of the project and the best way to propose and implement an increase in SSB taxes.

We are also proposing to work with Bloomberg Philanthropies in the development of a comprehensive Implementation Plan which will include key sectors and stakeholders involved in SSB production and consumption

The information generated through this project will be useful for policy development targeting SSB consumption. Policy change is a highly complex process and is shaped by a multitude of interacting forces and actor; this makes it difficult to predict the consequences of our projected activities. This proposal is innovative because we will address SSB consumption from different perspectives. From a structural perspective we will describe the SSB market process from the production of sugar to the selling of SSB to individual consumers. This information will allow us to explore multiple targets as possibly amenable to policy interventions while considering a broad array of policy arguments, including debates on worker's rights and environmental protection. From a legal perspective we will explore the regulations and legal framework of the SSB market and examine legal arguments that could be used to implement policies that guarantee the right of the population to receive healthy food and the role of government in ensuring that this happens. The economic perspective will explore the feasibility and the impact that potential rises in taxes will have on SSB consumption and what mechanisms within Argentina would enable these taxes to increase. Finally, using a public health perspective, we will define and explore the magnitude of cardiovascular mortality benefit associated with implementing policies aimed that reduce SSB consumption. The multidisciplinary team will work with consultants from Mexico and the US who will share their experience in generating similar evidence and in implementing policies. An international perspective will also help situate the Argentine experience into a broader context, rendering it potentially more useful to other countries in the region.

We expect that at the end of the project, we will have developed a **Policy Influence Plan** that will be able to be implemented in the short term. The implementation of this plan will result in new and important policy initiatives and this strategy could be implemented in other countries of the region.

Key partners and key milestones

Potential Partners	Possible Outcomes	Process Measures
Centro de Estudios Sobre Nutrición Infantil (CESNI)NGO focused on Child Welfare issues and policies. www.cesni.org.ar	Incorporation of obesity as a key focus in health	Ongoing dialogue on SSB consumption policies in marketplace and schools
Fundación Interamericana del Corazón Argentina (FIC Argentina) NGO focused on the promotion of public policies and social changes that guarantee the protection of the right to health, through the reduction of chronic non-communicable diseases. www.ficargentina.org	Incorporate SSB into their advocacy plan and their initiative on food labeling	Ongoing dialogue on SSB contribution to cardiac disease and potential policies to curb consumption
Asociación Defensa de Consumidores y Usuarios Argentina (Adecua), NGO focused on consumer rights. www.adecua.org.ar	Create awareness on rights of consumers to a healthy diet; incorporation of SSB/diet into their framework of consumer rights.	Ongoing engagement with coalition on SSB policies
Sociedad Argentina de Pediatria (SAP). Scientific Society of Pediatricians www.sap.org.ar	Include limitation of SSB consumption into SAP recommendations for children and adolescents	Engagement with scientific evidence base on SSB contribution to obesity and diabetes
Sociedad Argentina de Obesidd y Trastornos Alimentarios (SAOTA) Scientific society of experts in nutrition. http://www.saota.org.ar/ and Sociedad Argentina de Nutrición (SAN) www.sanutricion.org.ar	Include focus on SSB in their guidance and web materials	Engagement with scientific evidence base on SSB contribution to obesity and diabetes Engagement with policy debates
Health Comission at the National Congress	Meeting to discuss SSB contribution to chronic disease in Argentina	Ongoing engagement with policy options on taxation. Identification of legislative champions.
Ministry of Health	Include SSB into the Plan Nacional Argentina Saludable http://www.msal.gob.ar/argentina-saludable/plan/argsal.html	Ongoing engagement with policy options
Consejo Federal de Salud	Meeting to discuss SSB	Ongoing engagement

(COFESA);Ministry of Health http://www.msal.gob.ar/index.php/component/content/article/45-cofesa/32-cofesa	contribution to chronic disease in Argentina	with policy options.
Programa Nacional de Salud Escolar (PROSANE); Ministry of Health http://www.msal.gob.ar/index.php/programas-y-planes/229-programa-de-sanidad-escolar	Eliminate full sugar SSB in schools	Ongoing dialogue on SSB consumption policies in schools
Programa Nacional de Municipios y Comunidades Saludables; Ministry of Health http://www.msal.gob.ar/municipios/	Incorporation of obesity as a key focus in health	Ongoing engagement with policy options
Ministry of Health – City of Buenos Aires<br>Ministry of Health – Buenos Aires Province	Launch discussion of SSB policies in meeting reviewing the scientific evidence	Ongoing engagement with scientific evidence and policy options particularly school SSB policies.
Law Department at Universidad Torcuato Di Tella<br><br>Law School http://www.utdt.edu/ver_contenido.php?id_contenido=1336&id_item_menu=423<br><br>Right to health program http://www.utdt.edu/ver_contenido.php?id_contenido=10870&id_item_menu=21189	Launch discussion of the legal dimensions of SSB regulation and the right to health with key legal scholars and regulatory officials	Ongoing engagement with regulatory research and research to frame SSB's regulations as a right to health matter
UNICEF Argentina http://www.unicef.org/argentina/spanish	Incorporation of obesity as a key focus in health	Ongoing dialogue on SSB consumption policies in marketplace and schools

Research Capacity Building The overall goal of the training and capacity building is to instruct a new generation of researchers and policy leaders who will be active participants in the development and implementation of chronic disease policy oriented research, with particular expertise in SSB control. The training will also strengthen local institutions, so that they are able to augment the scientific skills of future generations of researchers. Because of the complex nature of nutrition and SSB consumption, specialized training in this field must combine substantive, methodological, and practical approaches to research. Cross-disciplinary information is needed to understand the forces that drive SSB use and its consequences. Therefore, a variety of research areas and fields of study are applicable to an understanding of SSB use including medical and

behavioral research, economics, health policy, communication, statistical analysis, legal analysis and political sciences. The unique nature of SSB-related research frequently requires collecting data from diverse groups of people, using both quantitative and qualitative methods, and working with federal, provincial, and local agencies. To successfully complete this research and at the same time build a foundation for future SSB-related projects, we propose to implement a multi-faceted training and capacity building component directed by Dr Fernandez and Dr Mejia. This training program will be based on mentorship as described above. In addition we´ll provide support for travel and training in the US to Dr. Maria Victoria Salgado to develop additional research skills on the CHD Policy Model.

Monitoring and evaluation plan

This exploratory project will be focused mainly on knowledge generation and the outputs will include academic articles, research reports, policy briefs, and a website of the project.

Each objective will have its own outputs and progress markers and they are described in the following table.

Specific Aim	Progress marker at Month 6	Progress marker at Month 12	Progress marker at Month 18	Final output
**#1: To develop a context map of the setting that surrounds SSB production, distribution and consum**	**Aim 1.1**. Comprehensive literature review about SSB production<br><br>Newspaper review<br><br>12 in-depth interviews completed	Preliminary report on sugar production and commercialization in Argentina during the last 20 years	Final report on sugar production and commercialization in Argentina during the last 20 years	Article and Policy brief of SSB production and consumption in Argentina
	**Aim 1.2** Comprehensive revision of literature about the stakeholders in SSB	18 in-depth interviews completed		Policy Network Map of SSB<br><br>Stakeholder Influence Map of SSB
	**Aim 1.3**		Survey to participants in the stakeholders workshop	Policy brief describing the force field analysis<br><br>Advocacy plan

**#2 Situation analysis of the national legal and regulatory framework**	**Part 1**. A document with the review of the national legal regulations	A Database with legal documents	Internal report on operation of a selection of accountability mechanisms	Legal Database on International, National and Provincial Laws regulating SSBs
	**Part 2**. Study of comparative SSBs regulatory events	Descriptive document about comparative regulatory events	Draft final report	Final report on regulations about SSBs
	**Part 3**. Normative research on grounds for SSBs' restrictive regulation	Literature review	Draft paper	Final scholarly paper on the human rights justification for the regulation of SSBs
**#3 Analysis of taxations**	Theoretical and Methodological Framework	Draft Stakeholder Analysis	Draft Complex System and Quantitative Analysis	Final report<br>Policy Brief<br>Academic article
**#4 Economic Analysis of Consumption**	Theoretical and Methodological Framework	Draft Descriptive Statistical Analysis	Draft Demand Elasticities	Final report<br>Policy Brief<br>Academic article
**# 5. Modeling the Impact of SSB consumption changes on Health Benefits and Health Care Costs**		Un updated version of the CHD Policy Model.<br>A scientific report describing the model updating		A scientific paper describing the simulation.

A Final report in English will be prepared for IDRC, and a Spanish version to be distributed among key stakeholders including the Ministry of Agriculture, the Ministry of Health, other partners of the project and institutions and organizations interested in reducing obesity and ensuring healthy food for the population.

Communications and knowledge translation plan

We will produce a series of policy briefs and scientific papers on the economic determinants of SSB consumption in Argentina, the regulation of sugar content in beverages; estimates of the benefits of a SSB consumption reduction via an SSB tax, and other public policies that could be implemented to reduce SSB consumption and their likely effects on the prevalence of cardiovascular diseases. The information obtained through this initial study will be presented in a format that will be easily accessible and understandable to key stakeholders in order to raise awareness of the harmful effects of SSB and stimulate discussion on for the importance of developing SSB policies at a national level.

The policy briefs produced will be distributed among decision makers from the Ministry of Health, Ministry of Productin, Ministry of Economy and Ministry of Agriculture, representatives of Food Industry (COPAL), and representatives of the civil society engaged in consumer's protection (CESNI and FIC Argentina); these evidence briefs will be discussed with diverse stakeholders in a workshop.

The information produced will be shared with other countries of the region through collaborations with PAHO to provide information for interventions in other countries beyond the scope of this grant.

The media will be included in the dissemination strategy to raise awareness among the general population on the harms of SSB intake. A media strategy will be coordinated through the CEDES information center. In addition, a web page will be developed for disseminating project results with other researchers and with the general population. The website will be placed in CEDES web site and will include a description of the research team, description of the project, all the outputs produced and a resource site for other researchers. We have developed websites for previous projects such as http://www.cedes.org/tabacoycine/el-problema.php

A set of academic articles will be prepared, one per objective, and submitted to peer reviewed journals under the "open access policy". These articles will make a significant contribution to the existing academic literature as SSB policies are a topic that is relatively understudied in Latin America.

Policies and actions to improve food systems for healthier diets need strong political commitment, combined with determined and sustained support from citizens and leadership from civil society organizations and social movements at all levels (national, state, and municipal).A cornerstone in the Knowledge Exchange plan will be the implementation of a 2 day workshop, coordinated by Ms Sarah Fine, in year 2. The list of participant to this workshop will include representatives from the Ministry of Health (ie,; National Director of Prevention of NCDs), from the Ministry of Economy, from the Ministry of Agriculture (Subsecretaria del valor agregado), Previous to this workshop participants will receive package with information of the results of the project this workshop; this information will be divided into general information with the principal findings and specific information, this last section will be tailored to the expertise of each attendee. We expect that at the end of the workshop participants will have a clear notion about the burden that SSB consumption carries to Public Health and will see the importance of acting immediately. Also, at the end of the workshop we´ll present a clear plan to make the change possible and we´ll make sure that there is a powerful group guiding the change. main outcome will be the inclusion of SSB in the agenda of the *Plan Argentina Saludable*, Ministry of Health, and we expect to start the new phase of the project with the development of robust policy dialogues on different SSB policies and regulations.

A complete report and an executive report will be prepared to IDRC.

Work plan and Schedule

Specific	Activity	Year 1	Year 2

Aim									
		Q1	Q2	Q3	Q4	Q1	Q2	Q3	Q4
#1	**1.1** Review of literature, press and statistics								
	**1.1** Complete in depth interviews								
	**1.1** Analysis of interviews								
	**1.1** Report on SSB consumption								
	**1.2** Complete 18 in depth interviews								
	**1.2** Policy Network Map of SSB								
	**1.2** Stakeholdesr Influence Map								
	**1.3** Designe and pilote survey								
	**1.3** Workshop with stakeholders								
	**1.3** Design advocacy plan								
	**Dissemination**								
#2	Legal data collection (Parts 1, 2 and 3)								
	Design of the legal database (Part 1)								
	Selection of accountability forums (Part 1)								
	Legal literature review) (Parts 1, 2 and 3)								
	Systematization of legal (Part 1)								
	Interviews with key informants (Part 1)								
	Legal database completion (Part 1)								
	Selection of regulatory episodes (Part 2)								
	Data collection on regulatory episodes								
	Interviews with key informants (Part 2)								
	Analysis of comparative experiences								
	Elaboration of final report and policy brief								
	Article with human rights framework								
	Dissemination of results (Parts 1, 2 and 3)								
#3	Literature Review - Theoretical Framework								
	Comparative Analysis								
	Stakeholder interviews								
	Complex System Analysis								
	Price Evolution Analysis								
	Draft Document Preparation								
	Final Products								
#4	Literature and Experiences Review								
	Database Building and descriptive analysis								
	Econometric Analysis - Demand Function								
	Econometric Analysis - Elasticities								
	Simulation Tax inclusions								
	Draft Document Preparation								
	Final Products								
#5	Data collection for updating the model								
	Consolidate the model.								
	Revise the model . Final version								
	SSB serving size calculation								
	Simulations Run								
	Paper writing, dissemination activities								

Ethical and community considerations

This project will comply with the ethical recommendations to preserve the identity of all persons to be interviewed. Al key informants will sign an informed consent form, that will be submitted to the CEMIC IRB for approval. The *Centro de Educación Medica e*

Investigación Clinica (CEMIC) Institutional Review Board, an internationally recognized Ethics Committee, will review our proposal. No subject contact will occur until approval has been obtained from CEMIC.

There are wide disparities in the distribution of CVD burden in Argentina that are partly explained by socioeconomic determinants. More specifically, clear gradients in self-reported health status, diet, obesity and diabetes have been observed by educational attainment and income, even after controlling for other independent variables(24, 46). The estimation of the long-term and short-term effects of prices on SSB demand and the affordability will provide information about the effect of rising process of SSB on low income people.

.

Profiles of applicant organizations and personnel

The research institution for this section of the proposal will be the Centro de Estudios de Estado y Sociedad (CEDES). Since its foundation in July 1975, the CEDES, a multidisciplinary institution, has focused on the study of social, political, and economic problems in Argentina and Latin America. Its mission is to foster research capability in the social sciences in Argentina and the rest of Latin America and the dissemination and application of its findings. In this way, the CEDES pursues to contribute to social and economic development and strengthen human rights and democracy as well as social and gender equity.

CEDES research products are disseminated through several channels: own publications, such as the series of CEDES studies and CEDES documents, and national and international books and periodicals. In addition, CEDES has its own information center where information on the thematic areas dealt with by the institution is gathered, organized and disseminated, with special emphasis on the field of social sciences applied to health. By means of its cooperative activities, it interacts directly with networks of national and international information sources to share resources and exchange information.

CEDES will provide Dr Mejía and his team with office space for meetings and administrative management of the project and administrative management of the budget. Finally, CEDES through its Dissemination, Advocacy and Network Development Program will disseminate the research findings to different stakeholders including officers of the Ministry of Health, public health practitioners within local public health units, local, regional, provincial and national offices of non-governmental organizations involved in providing healthy food, decision-makers including politicians, and journalists.

Researchers from the Instituto Nacional de Salud Publica de Mexico (INSP) will participate at an individual basis serving as consultants for the project. The INSP has a successful history of providing key research evidence that led to the implementation of an SSB tax in Mexico. They will participate in developing the models to transform the

calories from SSB in weight and the increase in weight in risk of diabetes, and in the design of the Policy Influence Map

Researchers from the SFGH –DGIM at the University of California, San Francisco, (UCSF) will also participate on an individual basis. The SFGH-DGIM groups multiple investigators at UCSF and is nationally and internationally known for its research in health communication and health policy to reduce health disparities, with special expertise in the social determinants of health, including literacy, food policy, poverty, and minority status, with a focus on the clinical conditions of pre-diabetes, diabetes, and cardiovascular disease.

Raul Mejia, MD, Phd, (PI), is an expert in the epidemiology and risk factors of cardiovascular disease. He has served as PI in research projects funded by IDRC and by Fogarty/NIH. He is Senior Researcher at CEDES. Dr. Mejia heads an active research team and has mentored over 30 junior researchers over his 20 years as a senior investigator. Dr. Mejia will be the PI at CEDES and will coordinate the articulation of the different objectives; he will coordinate the data collection and the interviews for the context mapping of SSB. He will devote 23% of his time to the project.

Alicia Fernandez, MD, (Consultant and Co PI) is a professor of medicine at the University of California San Francisco (UCSF). Her research focuses on chronic disease health care disparities with an emphasis on diabetes. Her research has been supported by the NIH and through foundation grants. Dr. Fernandez has been involved with analyses using the Mexico version of the Coronary Heart Disease (CHD) Policy Model evaluating the health impact of public health interventions in Mexico (Fogarty PI) and also collaborates with investigators in Argentina on projects using the Argentine version of the CHD Policy Model. Dr. Fernandez is a member of the IOM Roundtable on Literacy and was recently appointed to the Board of Governors of the Patient Centered Research Outcomes Institute (PCORI). She has been affiliated with CEDES since 2013. Dr. Fernandez will work with Dr. Mejia on all aspects of the Project, focusing particularly on mentorship of Dr. Salgado and Dr. Konfino, and broad dissemination of findings.

Paola Bergallo, LL.B., LL.M , J.S.M. and J.S.D is a law professor at the Universidad de Palermo and an associate researcher at the CONICET (Argentina). She has been a visiting professor in universities throughout the Americas and Europe. Her main areas of work are public law, health and human rights, and empirical legal studies. She has participated in research projects for the UN Fund for Population, the Pan-American Health Organization, the Ford Foundation and the Nordic Trust Fund for the World Bank, and the Chr. Michelsen Institute where she is a Global Fellow of the Center for Law and Social Transformation. She will devote 7% of her time to leading the analysis of relevant laws and regulations on SSB and will work with three research assistants (one will be selected next March):

Sonia Ariza is a law professor and researcher at Universdad de Palermo. She graduated as a lawyer from the Universidad del Externado in Colombia and pursued a master program in Constitutional Law and Human Rights at the Universidad de Palermo, where she expects to defend her thesis in 2016. Sonia Ariza coordinates the Programa de Investigación y Abogacía Feminista at Universidad de Palermo. She has worked as a

research assistant and consultant for projects of the Centro de Estudios de Estado y Sociedad (CEDES), the UN Fund for Population, the Nordic Trust Fund of the World Bank, International Planned Parenthood Federation, and the Ministry of Health of Argentina. She has taught and written on law and health, women's human rights, and adolescent's rights. Sonia Ariza is fluent in English and Spanish.

Cecilia Garibotti is a researcher at the School of Law of the Universidad de Palermo. She is a lawyer graduated with honors from the Universidad de Buenos Aires and a political scientist graduated from Universidad de San Andrés. She has received fellowships from Universidad de San Andrés and the Fulbright Foundation. Cecilia Garibotti specializes in public and administrative law. She has worked as an associate lawyer at the administrative law department of M. & M. Bomchill and has been a lawyer at the Gas Regulatory Agency of Argentina. She has written on public law and administrative regulation. She is fluent in English and Spanish.

Daniel Maceira, Ph.D. Senior Researcher CEDES, Independent Researcher CONICET(Argentina), and Professor at the University of Buenos Aires. Maceira has collaborated with a wide array of institutions: IDRC, UNICEF, WHO, PAHO, ECLAC, UNDP, USAid, IAF, Gates Foundation, The World Bank, GDN, the Global Fund; IADB, GAVI Alliance, among others. He has numerous peer review publications, and coordinated many research projects and technical assistance initiatives in developing countries. Since 2012 participates as the Latin American counterpart at the Program Working Group of Health Systems Global. Since 2014 he is academic co-cordinator of the Postgraduate Program on Health Management organized by FLACSO/Plan Sumar (Ministry of Health), Argentina. He will devote 14% of his time to the project and will lead the economic analysis with two research assistants:

Alfredo Palacios has a bachelor degree in Economics from the National University of Río Cuarto, and a Master in Economics from the National University of La Plata (thesis defense in process). It has also taken specialization courses at the National Institute of Public Health of Mexico and at the Institute for Development, Growth and Economics of Uruguay. His research interests focus on Applied Microeconometrics, Quantitative Methods of Impact Evaluation of Public Policy and Health Economics. Currently he is Assistant Researcher at the Center for the Study of State and Society (CEDES), Professor of Applied Econometrics in the economics graduate program at the National University of Lomas de Zamora, and Industrial Organization at the University of Buenos Aires (UBA). He has been consultant and / or collaborator in research projects for the World Bank, IADB, UNDP, World Health Organization.

Natalia Espinola is a Master in Economics from the National University of La Plata, Buenos Aires, Argentina. She is assistant professor and researcher at School of Economics, National University of La Plata, Argentina since 2013, and research assistant at Centro de Estudios de Estado y Sociedad (CEDES), Argentina. She works as a consultat and/or collaborator in research projects for the World Bank, Inter-American Development Bank, Ministry of Health of Argentina. Currently, she is working on a research project on institutional strengthening and stakeholder analysis of a

national health program. She has researched and written on stakeholder mapping, institutional assessment, institutional capacity building, health economics, microeconomics.

Jonatan Konfino MD: Masters in Clinical Effectiveness. He received training in public health, at the University of Buenos Aires, University of Toronto, and Johns Hopkins Bloomberg. He served as a fellow of the Research Training Program on Tobacco Control in Argentina. He is currently the National Directorate of Risks and Diseases Prevention at the National Ministry of Health. He will collaborate in the development of a context map of the setting that surrounds SSB market and he will collaborate with the analysis using the CVD Policy Model at 8% of his time.

Daniel Ferrante, MD, Msc is cardiologist and epidemiologist and coordinates the National Cardiovascular Prevention Program at the Ministry of Health. He has coordinated health promotion and population based surveillance initiatives, and has developed population modeling in the non-communicable disease and risk factors area. He has worked at WHO in the chronic disease cluster in Geneva. He will work 8% with Dr Salgado in the update of the CHD Policy Model and in modeling the effect of reducing SSB consumption.

Tamar Finzi, Sociologist, University of Buenos Aires, Master in Public Administration and Public Policies, Universidad Autónoma de Barcelona and Pompeu Fabra. She works at CEDES in the area of Health, Economy and Society since 2010. She participated in projects of applied investigation and consulting for the United Nations and national and municipal bodies. She participated in studies of the right to housing in Buenos Aires. In Barcelona she worked as researcher at the Institute of Government and Public Policies in the University of Barcelona (IGOP). She is member of the NGO Metodos, Interacción, Relevamiento y Análisis (MIRA). She will devote 15% of her time to conduct the context mapping section of the project. She will work with two research assistants (one will be selected next March):

Lorena Peña Sociologist, Buenos Aires University (UBA) 2008. Specialization in Epidemiology, Lanús University (UNLA) 2012. Student of Masters in Gender Studies, Business and Social Sciences University (UCES) 2014. Assistant researcher in health at CEDES. Has participated in an interdisciplinary team in projects on cultural meanings, knowledge and practices related to cancer, National Cancer Institute (INC), National Ministry of Health; and in the Project Tobacco Control in South America, Fogarty NIh.. Nowadays, works in the project Cinema Smoking and Youth Smoking in South America, funded by Fogarty/NIH under Dr mejia direction.

Victoria Salgado, MD, is a PhD student at Buenos Aires University, has worked on NCDs at Buenos Aires University and UCSF, publishing several articles on tobacco and young populations. In 2014 she participated as a teaching assistant in a course on policy-oriented research for prevention of cardiovascular disease, organized by UCSF and Mexico's Institute of Public Health. Dr. Salgado will be in charge of modelling the effect the consumption of SSBs has on cardiovascular disease morbidity and mortality using the CHD policy model, and projecting the health benefits of a reduction in SSBs intake associate with different price increases. She will devote 20% of her time.

Amalie Ablin: Obtained a degree in Political Sciences at the Universidad del Salvador, and a Degree in Agricultural Business at the University of San Andrés, finally completed a Master's in Agricultural Business at the Universidad Católica. Ms Ablin is an expert in and agro-industrial products, she has worked for the last 10 years at Ministry of Agriculture conducting analysis of the market for beverages -both alcoholic and non-alcoholic. She is recognized as a regional expert in SSB and published several articles in specialized journals. She will serve as consultant for the stakeholders mapping in the first year and will participate in the force field analysis and in designing the advocacy strategy during the second year of the project.

Sarah Fine has over ten years of experience in health communication and policy, media, program management, youth engagement, and the nonprofit sector. Her expertise lies in designing and implementing innovative, scalable, and high impact programs, and in building effective coalitions among diverse stakeholders. Sarah has worked to advance social initiatives both locally and internationally, from education advocacy in Argentina to public health equity in Nicaragua, Bolivia and across her home state of California. Sarah serves on the Diabetes Coalition of California's Advisory Board and oversees multiple projects for UCSF's Center for Vulnerable Populations' Health Communication Program at San Francisco General Hospital. She will contribute her expertise in Argentine coalition building to the project.

Risk mitigation and sustainability

One of the risks for that threat this project is that Argentina's the President and the Cabinet have changed parties in December 2015. While this will likely have consequences in the public health work, we expect the risks to this project to be manageable. We have a successful track in conducting research projects with changes in governmental authorities during the research period and we have the skills to include multiple viewpoints in the policy dialogues. Additionally, we expect there will be continuity at the MOH in key personnel who have expressed interest or are actively collaborating on this project. The current authorities of the MoH are is interested in this project because they were working at the MoH such as Sebastian Laspiur (Director for Prevention of NCDs) has expressed interest and Daniel Ferrante, a co-investgator will maintain their MOH positions. e, and they mentioned their interest in the project. In adition, Finally, Dr Zulma Ortiz, previous executive of UNICEF, is the current ministry of Health of Buenos Aires Province; Dr Ortiz has a large experience in prevention of NCDs and has worked in the past with Dr Mejia; , she has declared manifested her willingness to collaborate with the project. Another possible risk would be the potential constraint of geographical distance between partners. However, all project investigators have extensive experience with such collaborations, and the communication system used in previous projects will minimize distance barriers.

Finally, we cannot ignore the possible interference of SSB producers. In this exploratory phase of the project they might try to hinder access to the data needed for analysis. However, in the past we were very successful in gathering information about tobacco despite the lack of cooperation of tobacco producers so we do not believe that this would constitute a major drawback.

1. Basu S, Yoffe P, Hills N, Lustig RH. The relationship of sugar to population-level diabetes prevalence: an econometric analysis of repeated cross-sectional data. PloS one. 2013;8(2):e57873. PubMed PMID: 23460912. Pubmed Central PMCID: 3584048.
2. Babey SH, Jones M, Yu H, Goldstein H. Bubbling over: soda consumption and its link to obesity in California. Policy brief. 2009 Sep(PB2009-5):1-8. PubMed PMID: 19768858.
3. Woodward-Lopez G, Kao J, Ritchie L. To what extent have sweetened beverages contributed to the obesity epidemic? Public Health Nutr. 2011 Mar;14(3):499-509. PubMed PMID: 20860886.
4. Basu S, McKee M, Galea G, Stuckler D. Relationship of Soft Drink Consumption to Global Overweight, Obesity, and Diabetes: A Cross-National Analysis of 75 Countries. Am J Public Health. 2013 Mar 14. PubMed PMID: 23488503.
5. Xi B, Huang Y, Reilly KH, Li S, Zheng R, Barrio-Lopez MT, et al. Sugar-sweetened beverages and risk of hypertension and CVD: a dose-response meta-analysis. The British journal of nutrition. 2015 Mar 14;113(5):709-17. PubMed PMID: 25735740.
6. Malik VS, Schulze MB, Hu FB. Intake of sugar-sweetened beverages and weight gain: a systematic review. The American journal of clinical nutrition. 2006 Aug;84(2):274-88. PubMed PMID: 16895873. Pubmed Central PMCID: 3210834.
7. Fung TT, Malik V, Rexrode KM, Manson JE, Willett WC, Hu FB. Sweetened beverage consumption and risk of coronary heart disease in women. The American journal of clinical nutrition. 2009 Apr;89(4):1037-42. PubMed PMID: 19211821. Pubmed Central PMCID: 2667454.
8. Malik VS, Popkin BM, Bray GA, Despres JP, Hu FB. Sugar-sweetened beverages, obesity, type 2 diabetes mellitus, and cardiovascular disease risk. Circulation. 2010 Mar 23;121(11):1356-64. PubMed PMID: 20308626. Pubmed Central PMCID: 2862465.
9. Pan American Health Organization. Ultra-processed food and drink products in Latin America: Trends, impact on obesity,

policy implications. Washington, DC: PAHO, 2015.
10. Pan American Health Organization. PLAN DE ACCIÓN PARA LA PREVENCIÓN DE LA OBESIDAD EN LA NIÑEZ Y LA ADOLESCENCIA. In: PAHO, editor. 66a SESIÓN DEL COMITÉ REGIONAL DE LA OMS PARA LAS AMÉRICAS; Washington DC2014.
11. Bibbins-Domingo K, Coxson P, Pletcher MJ, Lightwood J, Goldman L. Adolescent overweight and future adult coronary heart disease. N Engl J Med. 2007 Dec 6;357(23):2371-9. PubMed PMID: 18057339.
12. Wang YC, Coxson P, Shen YM, Goldman L, Bibbins-Domingo K. A penny-per-ounce tax on sugar-sweetened beverages would cut health and cost burdens of diabetes. Health affairs. 2012 Jan;31(1):199-207. PubMed PMID: 22232111.
13. Mekonnen TA, Odden MC, Coxson PG, Guzman D, Lightwood J, Wang YC, et al. Health benefits of reducing sugar-sweetened beverage intake in high risk populations of California: results from the cardiovascular disease (CVD) policy model. PloS one. 2013;8(12):e81723. PubMed PMID: 24349119. Pubmed Central PMCID: 3859539.
14. Barquera S, Campirano F, Bonvecchio A, Hernandez-Barrera L, Rivera JA, Popkin BM. Caloric beverage consumption patterns in Mexican children. Nutrition journal. 2010;9:47. PubMed PMID: 20964842. Pubmed Central PMCID: 2987771.
15. Barquera S, Campos I, Rivera JA. Mexico attempts to tackle obesity: the process, results, push backs and future challenges. Obesity reviews : an official journal of the International Association for the Study of Obesity. 2013 Nov;14 Suppl 2:69-78. PubMed PMID: 24103026.
16. Brownell KD, Farley T, Willett WC, Popkin BM, Chaloupka FJ, Thompson JW, et al. The public health and economic benefits of taxing sugar-sweetened beverages. N Engl J Med. 2009 Oct 15;361(16):1599-605. PubMed PMID: 19759377. Pubmed Central PMCID: 3140416.

17. Ministerio de Salud de la Nación. ESTADISTICAS VITALES - INFORMACION BASICA AÑO 2008. In: DIRECCION DE ESTADISTICAS E INFORMACION DE SALUD, editor.: http://www.deis.gov.ar/publicaciones/archivos/Serie5Nro52.pdf; 2009.
18. Ministerio de Salud de la Nación. 3º Encuesta Nacional de Factores de Riesgo, Presentación de los principales resultados. Buenos Aires, Argentina: Ministerio de Salud http://www.msal.gov.ar/images/stories/publicaciones/pdf/11.09.2014-tercer-encuentro-nacional-factores-riesgo.pdf, 2014.
19. Konfino J, Martinez E, Ferrante D, Mejía R. Determinación de metas de factores de riesgo para enfermedades no transmisibles para 2016. Revista Argentina de Salud Pública. 2013;4:7-11.
20. Linetzky B, De Maio F, Ferrante D, Konfino J, Boissonnet C. Sex-stratified socio-economic gradients in physical inactivity, obesity, and diabetes: evidence of short-term changes in Argentina. International journal of public health. 2013 Apr;58(2):277-84. PubMed PMID: 22615030.
21. Rubinstein A, Gutierrez L, Beratarrechea A, Irazola VE. Increased prevalence of diabetes in Argentina is due to easier health care access rather than to an actual increase in prevalence. PloS one. 2014;9(4):e92245. PubMed PMID: 24699429. Pubmed Central PMCID: 3974703.
22. Ablin A, Naso MP. El mercado de bebidas analcohólicas. Alimentos Argentinos. 2012;54.
23. Ministerio de Salud de la Nación. Ecuesta Mundial de Salud Escolar 2007 Buenos Aires, Argentina2008 [cited 2013 April 21]. Available from: http://www.msal.gov.ar/tabaco/images/stories/info-equipos-de-salud/pdf/encuesta-mundial-salud-escolar.pdf.
24. Ferrante D, Linetzky B, Ponce M, Goldberg L, Konfino J, Laspiur S. Prevalence of overweight, obesity, physical activity and tobacco use in Argentine youth: Global School-Based Student Health Survey and Global Youth Tobacco Survey, 2007-2012. Archivos argentinos de pediatria. 2014 Dec;112(6):496-503. PubMed PMID: 25362906.
25. Konfino J, Ferrante D, Mejia R, Coxson P, Moran A, Goldman L, et al. Impact on cardiovascular disease events of the implementation of Argentina's national tobacco control law. Tobacco control. 2014 Mar;23(2):e6. PubMed PMID: 23092886. Pubmed Central PMCID: 4026283.
26. Ferrante D, Konfino J, Mejia R, Coxson P, Moran A, Goldman L, et al. [The cost-utility ratio of reducing salt intake and its impact on the incidence of cardiovascular disease in Argentina]. Rev Panam Salud Publica. 2012 Oct;32(4):274-80. PubMed PMID: 23299288. Relacion costo-utilidad de la disminucion del consumo de sal y su efecto en la incidencia de enfermedades cardiovasculares en Argentina.
27. Konfino J, Mekonnen TA, Coxson PG, Ferrante D, Bibbins-Domingo K. Projected impact of a sodium consumption reduction initiative in Argentina: an analysis from the CVD policy model--Argentina. PloS one. 2013;8(9):e73824. PubMed PMID: 24040085. Pubmed Central PMCID: 3767589.
28. Elorriaga N, Irazola VE, Defago MD, Britz M, Martinez-Oakley SP, Witriw AM, et al. Validation of a self-administered FFQ in adults in Argentina, Chile and Uruguay. Public Health Nutr. 2015 Jan;18(1):59-67. PubMed PMID: 24476763.
29. Cole DC, Johnson N, Mejia R, McCullough H, Turcotte-Tremblay AM, Barnoya J, et al. Mentoring health researchers globally: Diverse experiences, programmes, challenges and responses. Global public health. 2015 Aug 3:1-16. PubMed PMID: 26234691.
30. World Health Organization. 2008-2013 Action Plan for the Global Strategy for the Prevention and Control of Noncommunicable Diseases. Geneva: WHO, 2008.
31. Bogart W A. Regulating obesity?: government, society, and questions of health. New York: Oxford University Press; 2013.
32. Swinburn B, Kraak V, Rutter H, Vandevijvere S, Lobstein T, Sacks G, et al. Strengthening of accountability systems to create healthy food environments and reduce global obesity. Lancet. 2015 Jun 20;385(9986):2534-45. PubMed PMID: 25703108.

33. Jewell J, Hawkes C, Allen K WI. Law and obesity prevention: addressing some key questions for the public health community. http://www.wcrf.org/sites/default/files/WCRF-International-Law-and-Obesity-Prevention.pdf: World Cancer Research Fund International, 2013.
34. Laffont JJ, Tirole J. A Theory of Incentives in Procurement and Regulation. Boston, MA: MIT Press; 1993.
35. McFadden D. Conditional Logit Analysis of Qualitative Choice Behavior. In: P Z, editor. Frontiers of Econometrics. New York: Academic press; 1974.
36. Berry S. Estimating Discrete-Choice Models of Product Differentiation. RAND Journal of Economics. 1994;25(2).
37. Moran A, Coxson P, Ferrante D, Konfino J, Mejía R, Fernandez A, et al. The Cardiovascular Disease Policy Model: Using a National Cardiovascular Disease Simulation Model to Project the Impact of National Programs to Lower Dietary Salt. In: Legetic B, Cecchini M, editors. Applying Modeling to Improve Health and Economic Policy Decisions in the Americas: The Case of Noncommunicable Diseases. Washingtong DC: Pan American Health Organization,; 2015.
38. Bibbins-Domingo K, Coxson P, Pletcher MJ, Lightwood J, Goldman L. Adolescent overweight and future adult coronary heart disease. N Engl J Med. 2007;357(2371-9).
39. Weinstein MC, Coxson PG, Williams LW, Pass TM, Stason WB, Goldman L. Forecasting coronary heart disease incidence, mortality, and cost: the Coronary Heart Disease Policy Model. Am J Public Health. 1987;77:1417-26.
40. Moran A, Degennaro V, Ferrante D, Coxson PG, Palmas W, Mejia R, et al. Coronary heart disease and stroke attributable to major risk factors is similar in Argentina and the United States: The Coronary Heart Disease Policy Model. Int J Cardiol. May 6. PubMed PMID: 21550675. Epub 2011/05/10. Eng.
41. Rubinstein AL, Irazola VE, Calandrelli M, Elorriaga N, Gutierrez L, Lanas F, et al. Multiple cardiometabolic risk factors in the Southern Cone of Latin America: a population-based study in Argentina, Chile, and Uruguay. Int J Cardiol. 2015 Mar 15;183:82-8. PubMed PMID: 25662056. Pubmed Central PMCID: 4382451.
42. Schulze MB, Manson JE, Ludwig DS, Colditz GA, Stampfer MJ, Willett WC, et al. Sugar-sweetened beverages, weight gain, and incidence of type 2 diabetes in young and middle-aged women. JAMA. 2004 Aug 25;292(8):927-34. PubMed PMID: 15328324.
43. Imamura F, O'Connor L, Ye Z, Mursu J, Hayashino Y, Bhupathiraju SN, et al. Consumption of sugar sweetened beverages, artificially sweetened beverages, and fruit juice and incidence of type 2 diabetes: systematic review, meta-analysis, and estimation of population attributable fraction. BMJ. 2015;351:h3576. PubMed PMID: 26199070. Pubmed Central PMCID: 4510779.
44. Hall KD, Sacks G, Chandramohan D, Chow CC, Wang YC, Gortmaker SL, et al. Quantification of the effect of energy imbalance on bodyweight. Lancet. 2011 Aug 27;378(9793):826-37. PubMed PMID: 21872751. Pubmed Central PMCID: 3880593.
45. Ministerio de Salud de la Nación. Plan Argentina Saludable 2010 [cited 2010 31/7]. Available from: http://www.msal.gov.ar/argentina_saludable/plan/argsal.html.
46. De Maio FG, Konfino J, Ondarsuhu D, Goldberg L, Linetzky B, Ferrante D. Sex-stratified and age-adjusted social gradients in tobacco in Argentina and Uruguay: evidence from the Global Adult Tobacco Survey (GATS). Tobacco control. 2014 Jul 1. PubMed PMID: 24985731.
