# Supplementary material for: Projected impact of a reduction in sugar-sweetened beverage consumption on diabetes and cardiovascular disease in Argentina: A modeling study
Source: PLoS Med. 2020 Jul 28;17(7):e1003224. doi: 10.1371/journal.pmed.1003224 (PMC7386620; doi:10.1371/journal.pmed.1003224)
Supplement: S1 STROBE Checklist — STROBE, Strengthening the Reporting of Observational Studies in Epidemiology. (DOCX) [file pmed.1003224.s005.docx]

“Projected impact of a reduction in sugar-sweetened beverage consumption on diabetes and cardiovascular disease in Argentina: a modeling study”

by M. Victoria Salgado; Joanne Penko; Jonatan Konfino; Pamela G Coxson; Kirsten Bibbins-Domingo; Alicia Fernandez; Raul Mejia.

STROBE Statement—checklist of items that should be included in reports of observational studies

|  | | **Item No.** | | **Recommendation** | **Section/ Paragraph No.** | | | | **Relevant text from manuscript** | |  |  |  |
| --- | --- | --- | --- | --- | --- | --- | --- | --- | --- | --- | --- | --- | --- |
| **Title and abstract** | | 1 | | (*a*) Indicate the study’s design with a commonly used term in the title or the abstract | Title | | | | “Projected impact of a reduction in sugar-sweetened beverage consumption on diabetes and cardiovascular disease in Argentina: a modeling study” | |  |  |  |
|  |  |  |  | (*b*) Provide in the abstract an informative and balanced summary of what was done and what was found | Abstract – Paragraph 1 | | | | “We used the Cardiovascular Disease Policy Model (…) to estimate…” | |  |  |  |
| **Introduction** | |  | |  |  | | | |  | |  |  |  |
| Background/rationale | | 2 | | Explain the scientific background and rationale for the investigation being reported | Introduction, paragraphs 1 to 3 | | | |  | |  |  |  |
| Objectives | | 3 | | State specific objectives, including any prespecified hypotheses | Introduction, paragraph 4 | | | | “…we used (CVD) Policy Model (…) to simulate the impact of reduced SSB consumption on national diabetes incidence, cardiovascular events and mortality” | |  |  |  |
| **Methods** | |  | |  |  | | | |  | |  |  |  |
| Study design | | 4 | | Present key elements of study design early in the paper | Methods – Section: CVD Policy Model-Argentina | | | |  | |  |  |  |
| Setting | | 5 | | Describe the setting, locations, and relevant dates, including periods of recruitment, exposure, follow-up, and data collection | Not completely applicable to a modeling study, although sources of information were all described in the Methods section | | | |  | |  |  |  |
| Participants | | 6 | | (*a*) *Cohort study*—Give the eligibility criteria, and the sources and methods of selection of participants. Describe methods of follow-up  *Case-control study*—Give the eligibility criteria, and the sources and methods of case ascertainment and control selection. Give the rationale for the choice of cases and controls *Cross-sectional study*—Give the eligibility criteria, and the sources and methods of selection of participants | Not applicable to a modeling study | | | |  | |  |  |  |
|  |  |  |  | (*b*) *Cohort study*—For matched studies, give matching criteria and number of exposed and unexposed  *Case-control study*—For matched studies, give matching criteria and the number of controls per case | Not applicable to a modeling study | | | |  | |  |  |  |
| Variables | | 7 | | Clearly define all outcomes, exposures, predictors, potential confounders, and effect modifiers.  Give diagnostic criteria, if applicable | Methods – Section: Model Simulations. Paragraph 1 | | | | “We modeled the impact of a reduction in sugar-sweetened soda consumption on diabetes incidence, myocardial infarctions, strokes, CVD mortality, and all-cause mortality using the CVD Policy Model-Argentina over a 10-year period from 2015 to 2024” | |  |  |  |
| Data sources/ measurement | | 8* | | For each variable of interest, give sources of data and details of methods of assessment  (measurement). Describe comparability of assessment methods if there is more than one group | Methods – Sections: CVD Policy Model-Argentina - Estimating daily SSB consumption in Argentina - SSB effect on cardiovascular disease risk factors | | | |  | |  |  |  |
| Bias | | 9 | | Describe any efforts to address potential sources of bias | Methods – Section: Estimating daily SSB consumption in Argentina – Paragraph 2 and 3  Appendix S1 | | | |  | |  |  |  |
| Study size | | 10 | | Explain how the study size was arrived at | Not applicable to a modeling study | | | |  | |  |  |  |
| Quantitative variables | | 11 | | Explain how quantitative variables were handled in the analyses. If applicable, describe which groupings were chosen and why | Not applicable to a modeling study | | | |  | |  |  |  |
| Statistical methods | 12 | (*a*) Describe all statistical methods, including those used to control for confounding | | | Methods – Sections: CVD Policy Model-Argentina - SSB effect on cardiovascular disease risk factors | | |  | | | |  |  |
|  |  | (*b*) Describe any methods used to examine subgroups and interactions | | | Not applicable to a modeling study | | |  | | | |  |  |
|  |  | (*c*) Explain how missing data were addressed | | | Not applicable to a modeling study | | |  | | | |  |  |
|  |  | (*d*) *Cohort study*—If applicable, explain how loss to follow-up was addressed  *Case-control study*—If applicable, explain how matching of cases and controls was addressed *Cross-sectional study*—If applicable, describe analytical methods taking account of sampling strategy | | | Not applicable to a modeling study | | |  | | | |  |  |
|  |  | (*e*) Describe any sensitivity analyses | | | Methods – Section: Probabilistic Sensitivity Analyses | | | “We used Monte Carlo simulations to generate 95% uncertainty intervals (UIs) around our primary outcome measures for each intervention scenario.” | | | |  |  |
| **Results** |  |  | | |  | | |  | | | |  |  |
| Participants | 13* | (a) Report numbers of individuals at each stage of study—eg numbers potentially eligible, examined for eligibility, confirmed eligible, included in the study, completing follow-up, and analysed | | | Not applicable to a modeling study | | |  | | | |  |  |
|  |  | (b) Give reasons for non-participation at each stage | | | Not applicable to a modeling study | | |  | | | |  |  |
|  |  | (c) Consider use of a flow diagram | | |  | | |  | | | |  |  |
| Descriptive data | 14* | (a) Give characteristics of study participants (eg demographic, clinical, social) and information on exposures and potential confounders | | | Not applicable to a modeling study | | |  | | | |  |  |
|  |  | (b) Indicate number of participants with missing data for each variable of interest | | | Not applicable to a modeling study | | |  | | | |  |  |
|  |  | (c) *Cohort study*—Summarise follow-up time (eg, average and total amount) | | | Not applicable to a modeling study | | |  | | | |  |  |
| Outcome data | 15* | *Cohort study*—Report numbers of outcome events or summary measures over time | | | Results – Tables 2 and 3, Figures 1 and 2 | | |  | | | |  |  |
|  |  | *Case-control study—*Report numbers in each exposure category, or summary measures of exposure | | | Not applicable to a modeling study | | |  | | | |  |  |
|  |  | *Cross-sectional study—*Report numbers of outcome events or summary measures | | | Not applicable to a modeling study | | |  | | | |  |  |
| Main results | 16 | (*a*) Give unadjusted estimates and, if applicable, confounder-adjusted estimates and their precision (eg, 95% confidence interval). Make clear which confounders were adjusted for and why they were included | | | Results | | | In the methods section: “We used Monte Carlo simulations to generate 95% uncertainty intervals (UIs)…” | | | |  |  |
|  |  | (*b*) Report category boundaries when continuous variables were categorized | | |  | | |  | | | |  |  |
|  |  | (*c*) If relevant, consider translating estimates of relative risk into absolute risk for a meaningful time period | | |  | | |  | | | |  |  |
| Other analyses | 17 | Report other analyses done—eg analyses of subgroups and interactions, and sensitivity analyses | | | Results - Low and high estimates of consumption | |  | | | |  |  |  |
| **Discussion** |  |  | | |  | |  | | | |  |  |  |
| Key results 18 | | | Summarise key results with reference to study objectives | | | | Discussion – Paragraph 1 | | |  | |  |  |
| Limitations 19 | | | Discuss limitations of the study, taking into account sources of potential bias or imprecision. Discuss both direction and magnitude of any potential bias | | | | Discussion – Paragraph 4 | | | “Our study limitations are…” | | | |
| Interpretation 20 | | | Give a cautious overall interpretation of results considering objectives, limitations, multiplicity of analyses, results from similar studies, and other relevant evidence | | | | Discussion – Paragraph 2, 3, 5 | | |  | | | |
| Generalisability 21 | | | Discuss the generalisability (external validity) of the study results | | | | Not applicable to a modeling study | | |  | | | |
| **Other information** | | |  | | | |  | | |  | | | |
| Funding 22 | | | Give the source of funding and the role of the funders for the present study and, if applicable, for the original study on which the present article is based | | | | Stated during the submission process | | |  | | | |

*Give information separately for cases and controls in case-control studies and, if applicable, for exposed and unexposed groups in cohort and cross-sectional studies.

**Note:** An Explanation and Elaboration article discusses each checklist item and gives methodological background and published examples of transparent reporting. The STROBE checklist is best used in conjunction with this article (freely available on the Web sites of PLoS Medicine at http://www.plosmedicine.org/, Annals of Internal Medicine at http://www.annals.org/, and Epidemiology at http://www.epidem.com/). Information on the STROBE Initiative is available at www.strobe-statement.org.
