# Supplementary material for: Projected impact of a reduction in sugar-sweetened beverage consumption on diabetes and cardiovascular disease in Argentina: A modeling study
Source: PLoS Med. 2020 Jul 28;17(7):e1003224. doi: 10.1371/journal.pmed.1003224 (PMC7386620; doi:10.1371/journal.pmed.1003224)
Supplement: Attachment — Submitted filename: Salgado et al - response to editors-reviewers.docx [file pmed.1003224.s006.docx]

**“Projected impact of a reduction in sugar-sweetened beverage consumption on diabetes and cardiovascular disease in Argentina: a modeling study”**

**by M. Victoria Salgado; Joanne Penko; Jonatan Konfino; Pamela G Coxson; Kirsten Bibbins-Domingo; Alicia Fernandez; Raul Mejia.**

**(Manuscript Number: PMEDICINE-D-20-00333R1)**

**Response to the Editors’ Comments:**

Main comment:

1. As noted by one reviewer, addressing more fully the range of parameter uncertainty in the model would be helpful, although we recognize this would represent a substantial amount of reworking of the paper and presentation of the findings. However, we look forward to the authors' justification on this point, and if the authors choose not to address this in the way suggested by the reviewer, we feel a strong justification would be needed.

*We estimated uncertainty intervals and presented them in the Results section (pages 12-15, lines 248-296). We have also added a paragraph in the Methods section describing how they were calculated (page 12, lines 234-246). Please, see the response to Reviewer #1.*

*We would also wish to thank you for recognizing the amount of work this process have involved, and we agree with you in that computing uncertainty intervals greatly improves the paper.*

Other comments:

2. Please structure your abstract using the PLOS Medicine headings (Background, Methods and Findings, Conclusions-"Methods and Findings" should be a single subsection).

*Methods and Findings have been merged as a single subsection (Page 2, lines 28 and 38).*

3. At this stage, we ask that you include a short, non-technical Author Summary of your research to make findings accessible to a wide audience that includes both scientists and non-scientists. The Author Summary should immediately follow the Abstract in your revised manuscript. This text is subject to editorial change and should be distinct from the scientific abstract.

*We have included the Author Summary after the Abstract (Pages 4 and 5, lines 59-90).*

4. In the last sentence of the Abstract Methods and Findings section, please describe the main limitation(s) of the study's methodology.

*We have added the following phrase at the end of the Abstract’s Methods and Findings section: “**The main limitations of our study are the limited availability of SSB consumption data in Argentina, and the fact that we were only able to model the possible benefits of the interventions for the population older than 34 years” (Page 3, lines 42-54).*

**Response to Reviewer #1:**

1. I have an important suggestion that might improve the manuscript. In its current state, the modelling exercise does not take into account the uncertainty in the parameters of Table 1. For example, the effect on systolic blood pressure is 0.78 mmHg (95% CI 0.09 to 1.47), but the authors use 0.78 mmHg in the model, as shown on Figure S2. A more realistic modelling exercise would use Monte-Carlo simulations to assess the reduction in number of events by taking into account the uncertainty on all parameters of Table 1 (diabetes, fatal and non-fatal cvd events, mortality). Using Monte-Carlo simulations, the authors would be able to provide a range for their estimates (and present this visually using histograms) for the various scenarios (low vs high consumption, caloric compensation of 0%, 39% and 100%).

I recognise that my suggestion will generate more work for the authors. Nonetheless I think that providing uncertainty intervals for the estimates would noticeably improve the paper. For example, the sentence in the abstract that currently reads "a 10% reduction in SSB with a caloric compensation of 39% is projected to reduce incident diabetes cases by 13300 to 27700 cases compared to a scenario of no change in SSB consumption" would include uncertainty intervals around 13300 and 27700. Given the uncertainty in the effects of the intervention (as recognised in Table 1) it would be more appropriate to propagate the uncertainty in the modelling exercise.

*We have estimated uncertainty intervals for our outcomes, and agree in that their inclusion greatly improves the paper.*

*Uncertainty intervals are now not only presented in the Results section (pages 12-15, lines 248-296).; we also have added the following paragraph at the end of the Methods section (page 12, lines 234-246):*

*“Probabilistic Sensitivity Analyses*

*We used Monte Carlo simulations to generate 95% uncertainty intervals (UIs) around our primary outcome measures for each intervention scenario. The 95% confidence intervals for estimates of the effect of changes in SSB consumption on SBP and on diabetes risk as well as the beta inputs for model risk functions defining the relationship between risk factors and incident diabetes, incident CHD, incident stroke, and non-CVD death are included in Table S3 of the Appendix. There were 1,000 random draws from a standard normal distribution, scaled to the mean and confidence interval, for each varied parameter. The Monte Carlo program, written in Python, generated a new set of input parameters drawn from the distributions for each iteration, ran the given iteration base case and reduced SSB consumption simulations with the new parameters, and stored the outcomes for each iteration. The 95% UIs for each outcome were then calculated using Microsoft Excel 2016.”*

**Response to Reviewer #2:**

1. Abstract: The line before conclusions, please clarify at which age gender you refer when saying." Cardiovascular events occurred in the youngest age group (35-44 y)" It is confusing as you mentioned afterwards that the reduction would also occur in men all age.

*We have changed the phrase from “In all scenarios, the largest reductions in diabetes and cardiovascular events occurred in the youngest age group modeled (35–44 y), and in men, regardless of age” to “The largest reductions in diabetes and cardiovascular events occurred in the youngest age group modeled (35–44 y) for both men and women; additionally, more events were avoided in men compared to women in all age groups” (page 3, lines 49-51).*

**Response to Reviewer #3:**

1. They focused on CVD outcomes, due to the nature of the model they selected, which is only part of the global SSB public health picture. It is important to note that for SSBs as a health risk factor, the population of children, adolescents and young adults is the most affected and this is not adequately captured by this model. Also, other relevant public health outcomes such as dental caries, bullying, depression, cancer of many sites, and many medical conditions such as nonischemic heart disease, osteoarthritis, kidney disease, dementia and others, could be incorporated in future SSB-specific modeling. The discussion should have highlighted this wider panorama.

*We agree with you in that the age range covered by the CVD Policy Model is one of its limitations. Nevertheless, we feel that this issue has already been addressed in the limitations section of the discussion, stating that “Finally, we were only able to model the possible benefits for the population older than 34 years, -- the age range analyzed by the CVD Policy Model— and for a ten-year period. As most sodas are consumed by younger people, the health benefits of consumption reduction would be higher among younger generations over time.”*

*We completely agree that it is important to conduct analyses on the non-CVD health effects of SSB beverages. Unfortunately, due to the intrinsic nature of the CVD Policy Model, we are unable to do that in this study. Other models that focus on cancer, dental health, and dementia, for example, could better inform the impact of SSB on the non-CVD benefits of reductions in SSB intake.*

2. From the content point of view, what seems to be also missing is a clear and wider explanation of strengths/weaknesses, particularly in context of other concurrent measures to be taken in NCD prevention.

*The CVD policy model calculates risks of CVD death via detailed modeling of cardiovascular disease events (angina, AMI, arrest, stroke). Non-CVD death risk is calculated directly as a function of the same risk factors (SBP, LDL-c, HDL-c, smoking, Type 2 Diabetes, BMI), without details of the events that would mediate that effect, so this outcome is necessarily harder to interpret.*

*We agree that reduction of SSB consumption is just one of many CVD and non-CVD non-communicable disease prevention measures that should be considered.*

*For example, while we are estimating that a 10% reduction in SSB consumption would all-cause deaths by 2,700 to 5600 for “low” and “high” estimates of SSB intake, respectively; a 5 to 15% salt reduction in processed food in Argentina would be expected to avert about 19,000 overall deaths in a decade (Konfino J et al, PLoS One 2013).*

*To highlight this issue, the last phrase of our article currently states: “Argentina has previously used computer simulation research results to foster national policy development. For example, other modeling studies on tobacco control and salt consumption (using the CVD Policy Model–Argentina) highlighted the impact of potential policies that were subsequently implemented”.*

3. Ref11 is a short newspaper article, not suitable as a source for a manuscript in my opinion. Better to original sources. Euromonitor is referenced elsewhere in the manuscript.

*We have taken out the newspaper article as a reference, and included Euromonitor information as another reference for the involved statement (page 6, line 100).*

4. Ref 41 for the estimate of calorie compensation is rather old. doi: 10.1038/ijo.2015.177 This meta-analysis from Rogers et al could be helpful. However, the authors correctly considered two extreme cases to account for this.

*We agree that information on how exactly a decrease in caloric intake is compensated is scarce. Thank you for noticing that we simulated not only the 39% caloric compensation scenario, but also the extreme ones (0% and 100%) to address this issue.*

5. Table 1. Some relative risks (RRs) might have been more updated or better substantiated by evidence. For example, the Global Burden of Disease 2017 study, available from http://ghdx.healthdata.org/gbd-results-tool would be an alternative and excellent source for deriving updated RRs.

*As you point out, the Global Burden of Disease study is an excellent source of information. However, in this particular case, we prefer to rely on studies that specifically address the link between SSB consumption and CVD risk factors, as well as information coming from the Framingham study, another worldly recognize source of information when it comes to CVD risk factors and outcomes.*

6. S2 table: what are exactly MI Deaths and arrests? what ICD10 codes were considered? There is no mention of corrections for garbage codes, or under-reporting of CV deaths, although it seems to have been done, and stated in the Medicina companion paper (Ref 27), I think it is worth to mention this in the main manuscript and in the Supplementary file.

*In the appendix, we have changed the original phrase*

*“Initial event rates in the population with prevalent cardiovascular disease were assumed from the prior model version and were adjusted iteratively until the model predictions adjusted to the actual health statistics in 2010, the baseline year (S2 Table)”*

*to*

*“Initial event rates in the population with prevalent cardiovascular disease were assumed from the prior model version. For calibration of mortality outcomes, the actual total number of deaths attributable to coronary heart disease was estimated as a compound of both definite CHD deaths (codes I20-I25 of the International Classification of Diseases, 10th Revision) in health records plus a percentage of poorly defined deaths (named ‘garbage’ codes, already defined) that could be attributed to CHD deaths. Garbage codes are codes assigned to deaths that were supposedly misclassified as non-CHD deaths when they should have been coded as CHD deaths. The total number of deaths obtained was later corrected by a factor determined for countries with low quality of registry (such as Argentina) in the Global Burden of Disease initiative. A similar method was then used to compare predicted and reported stroke deaths (using codes I60-I69). Model predictions were then adjusted iteratively until they matched the actual health statistics in 2010, the baseline year (S2 Table)”. We added the corresponding references (Appendix page 5).*

7. BMI effects on CVD events independent of DBT and lipids are not considered, possibly making the model estimates even more conservative. See for example DOI: 10.1001/archinte.167.16.1720 This could be discussed.

*It is correct we modeled BMI effects through its impact on blood pressure, diabetes and cholesterol levels. And as mentioned, it is possible that BMI has an independent effect on CVD outcomes. But given that this independent effect is still being analyzed, and confounding factors still need to be fully addressed, we chose to take as conservative a position as possible and so chose not to include the potential independent role of BMI on Coronary Heart Disease.*

8. Also, the model applied changes in the first year and then assumes to hold constant which could be questionable.

*This is an important observation. The impact of a tax on SSB is predicted based on economic studies of elasticity of price. Holding the change in consumption constant is first of all simply an assumption that the tax would continue at the same level and therefore keep consumption at the lower rate. One variable that could change is caloric compensation, so the simulations that examined different levels of compensation are presented as alternatives, but they could also be viewed as bounds for the case where compensation varies (one might imagine that compensation could trend up or down over a period of time). Being currently in the middle of an unexpected pandemic reminds us of how volatile economic situations can change all that was assumed to be constant in a simulation. Simulations can never take into account all possible scenarios, and in fact, it is the “all other things being equal” assumption of a modeling exercise that help us to understand the primary influence of the factor under consideration.*

*We have added the following text to the limitations section to address this point: “Additionally, our main scenario assumes a 39% caloric compensation; this estimate comes from US data and may be higher or lower in Argentina or could vary in a systematic way over time. Also, the effect size and all model parameters are held constant, varying only by age as the population ages, which is both a strength and a limitation of modeling” (page 17, lines 335-337).*

9. The term 'diabetes' should be replaced by 'Type 2 Diabetes Mellitus' which is more accurate.

*The initial phrase in the introduction has been changed from “As one of the main sources of added sugar in Western diets, sugar-sweetened beverage (SSB) consumption is suggestive of poor dietary quality and is associated with obesity, diabetes and hypertension.” to “As one of the main sources of added sugar in Western diets, sugar-sweetened beverage (SSB) consumption is suggestive of poor dietary quality and is associated with obesity, type 2 Diabetes Mellitus (from now on, referred only as “diabetes”) and hypertension” (page 6, line 94).*

10. Table 2: it would be clearer to say CVD deaths instead of CVD mortality (which is a rate)

*Thank you – we have made the change in both Tables 2 and 3 (pages 13 and 14).*

11. Fig 2 almost unreadable, need to fix the resolution of the image.

*We believe the lack of clarity is due to the automatic transformation process into the manuscript draft PDF made by the submission site, which somehow decreases the quality of the figures. In the same PDF, you can find a link to download the figures as TIF files (upper right corner), which when opened have a much greater resolution.*
