# Supplementary material for: Projected impact of a reduction in sugar-sweetened beverage consumption on diabetes and cardiovascular disease in Argentina: A modeling study
Source: PLoS Med. 2020 Jul 28;17(7):e1003224. doi: 10.1371/journal.pmed.1003224 (PMC7386620; doi:10.1371/journal.pmed.1003224)
Supplement: Attachment — Submitted filename: Salgado et al - response to editor.docx [file pmed.1003224.s007.docx]

**“Projected impact of a reduction in sugar-sweetened beverage consumption on diabetes and cardiovascular disease in Argentina: a modeling study”**

**by M. Victoria Salgado; Joanne Penko; Alicia Fernandez; Jonatan Konfino; Pamela G Coxson; Kirsten Bibbins-Domingo; Raul Mejia.**

**(Manuscript Number: PMEDICINE-D-20-00333R2)**

**Response to the Editors’ Comments:**

1. Abstract needs additional background to provide context for the study

*We have changed Abstract’s Background section from:*

*“Argentina is one of the major consumers of sugar-sweetened beverages (SSB) worldwide. Determining the impact of SSB reduction on health will inform policy debates.”*

*to*

*“sugar-sweetened beverage (SSB) consumption is associated with obesity, diabetes and hypertension. Argentina is one of the major consumers of SSB per capita worldwide. Determining the impact of SSB reduction on health will inform policy debates” (page 2, lines 25-28).*

2. Some tempering of language in the abstract and throughout the submission- “would” and “were” should be changed to “could”, “occurred” should be changed to “observed” to reflect the study design and observational nature of your study

*We have made the suggested changes.*

3. Please add a space between text and reference square brackets followed by a full stop throughout.

*We have made the changes.*

4. Throughout the manuscript please avoid the use of stigmatising labels such as “overweight” or “obese” and instead use “unhealthy weight” and “with obesity”.

*We have made the suggested changes when the adjectives were in reference to people and not a weight category (i.e., when saying “overweight and obesity”) (page 6, lines 106 and 111).*

5. Line 111 please revise to “non-communicable” disease

*We have made the suggested change (page 6, line 115).*

6. Line 154, 158 “gender” should be revised to “sex”

*We have made the suggested change (page 8, lines 160 and 164).*

7. Did your study have a prospective protocol or analysis plan? Please state this (either way) early in the Methods section.

*We do have a prospective protocol, developed for our funding proposal. We have included it as an Additional information document (S3 Appendix: Prospective protocol from funding proposal) (page 26, line 564).*

*We have also added the following sentence in the Methods section:*

“*The study’s prospective protocol can be found in the S3 Appendix” (page 8, lines 154-155).*

8. Table 2,3- please add “projected” when you mention prevented as this is a modelling study. The same goes for figures and captions.

*We have made the suggested change in both tables and figures.*

9. Please ensure that the study is reported according to the STROBE guideline, and include the completed checklist as Supporting Information. When completing the checklist, please use section and paragraph numbers, rather than page numbers. Please add the following statement, or similar, to the Methods: "This study is reported as per the Strengthening the Reporting of Observational Studies in Epidemiology (STROBE) guideline (S1 Checklist)."

*We have included the STROBE Checklist as an Additional Information document (S2 Appendix). Given that ours is not a cohort, case-control or cross-sectional study, there were selected items that we remark as “Not applicable to a modeling study” (page 26, lines 562-563).*

*We have also included the following sentence at the end of the first subsection of the Methods section (CVD Policy Model-Argentina): “This study is reported as per the Strengthening the Reporting of Observational Studies in Epidemiology (STROBE)* *guideline (S2 Appendix)” (page 8, lines 156-157).*

10. it looks like the model has been published previously, but I'm not sure "contact the authors" in the data statement complies with our usual policy - that might be best removed.

*The phrase has been removed.*

11. "per capita" at line 26.

*“per capita” was added in the Abstract’s Background section (page 2, line 27).*

12. around line 40, it would be helpful to quote the projected percentage reduction in incident T2D cases, as at line 252

*We have included in the Abstract the projected percentage reduction for diabetes cases (page 2, line 44).*

13. At lines 56 and 85, suggest replacing "significant" with "substantial" (or similar).

*We have made the suggested change (page 3, line 60, and page 5, line 89).*

*.*

14. At line 76, "projected to decrease" is not quite grammatical (e.g., "projected to lead to a decrease").

*We have replaced “projected to decreased” with “projected to avoid” (page 4, line 79).*

15. "Discussion" at line 296.

At line 302 where the authors summarize the findings, they start by noting a conservative projected reduction of 5100 T2D cases, which I don't think is quoted in the abstract or author summary (it does appear in the results). I'd suggest they restructure this to avoid confusing readers

*In the abstract and the author summary, we have decided to include our main scenario of 39% caloric compensation. Nevertheless, the most conservative scenario, which would be the one with 100% caloric compensation, could lead to a reduction of 5100 cases of diabetes; but considering the limited nature of both abstract and author summary, we have chosen not to include this scenario in them.*

16. Introduce UI on first view in the abstract

*We have added the following sentence in the Abstract’s Methods and Findings section: “We used Monte Carlo simulations to generate 95% uncertainty intervals (UI) around our primary outcome measures for each intervention scenario” (page 2, lines 38-40).*
